# Gut bacterial diversity of the tribes of India and comparison with the worldwide data

**DOI:** 10.1038/srep18563

**Published:** 2015-12-22

**Authors:** Madhusmita Dehingia, Kanchal Thangjam devi, Narayan C. Talukdar, Rupjyoti Talukdar, Nageshwar Reddy, Sharmila S. Mande, Manab Deka, Mojibur R. Khan

**Affiliations:** 1 Molecular Biology and Microbial Biotechnology Laboratory, Life Science Division, Institute of Advanced Study in Science and Technology (IASST), An autonomous institute under Department of Science and Technology (Govt. of India), Paschim Boragaon, Garchuk, Guwahati-781035, Assam, India; 2 Institute of Bioresources & Sustainable Development (IBSD), An autonomous institute under Department of Biotechnology (Govt. of India), Takyelpat, Imphal-795001, Manipur, India; 3 Asian Healthcare Foundation/Asian Institute of Gastroenterology (AIG), 6-3-661, Somajiguda, Hyderabad-082, Telangana, India; 4 Head of Bio-Sciences R&D, TCS Innovation Labs, Tata Consultancy Services Ltd. 54-B, Hadapsar Industrial Estate, Pune-411013, Maharastra, India; 5 Head of Department of Applied Sciences, Gauhati University, Guwahati-781014, Assam, India

## Abstract

The gut bacteria exert phenotypic traits to the host but the factors which determine the gut bacterial profile (GBP) is poorly understood. This study aimed to understand the effect of ethnicity and geography on GBP of Mongoloid and Proto-Australoid tribes of India. Fecal bacterial diversity was studied in fifteen tribal populations representing four geographic regions (Assam, Telangana, Manipur and Sikkim) by DGGE followed by NGS analysis on Illumina MiSeq platform. Geography and diet had significant effect on GBP of the Indian tribes which was dominated by *Prevotella*. The effects were more prominent with lower taxonomic levels, indicating probable functional redundancy of the core GBP. A comparison with the worldwide data revealed that GBP of the Indian population was similar to the Mongolian population (Mongolia). The bacterial genera *Faecalibacterium*, *Eubacterium*, *Clostridium*, *Blautia*, *Ruminococcus* and *Roseburia* were found to be core genera in the representative populations of the world.

The gastrointestinal tract of human is inhabited by trillions of bacteria of diverse nature, composition of which varies among individuals within and between communities^1^,^2^,^3^,^4^. The initial inoculum is acquired maternally during birth^5^ or even in utero^6^. Subsequent colonization of the gut depends upon several factors including diet, age and diseases^7^,^8^,^9^. Although not properly understood, the bacterial communities exert phenotypic traits to the host by a complex network of interactions among them^10^,^11^,^12^. Many of such interactions arising from the altered gut bacterial profiles (GBP) have been implicated to cause diseases in human^13^. Moreover, modern lifestyle with altered GBP has been thought to cause inflammatory disorders in the western countries^14^. To understand GBP of the diseased state, knowledge on GBP in the healthy state is also important. The GBP of several populations of the world, both with modern and traditional lifestyles have been studied in America, Europe, Africa, Korea and China^7^,^15^,^16^,^17^. With its largest tribal population, India offers a unique scenario for such studies, since diverse communities are still dependent on hunting, agriculture and fishing with their own culture, tradition, dietary habits, language and genetic make-up^18^,^19^. Till now there is no such report on GBP of the ethnic tribes of India. With the rapid economic development coupled with modernization of the lifestyle, the tribal population of India may eventually undergo alteration in GBP causing a gap in our knowledge on GBP across the globe. The need of data on GBP of ethnic tribes has earlier been emphasized^18^,^20^.

This study was conducted to understand the GBP of diverse ethnic tribes of India and to compare their GBP with those of other populations of the world. The GBP of 193 volunteers (apparently healthy) from 15 ethnic tribes (Mongoloids and Proto-Australoids) of India (Fig. 1) representing four geographic regions were studied using 16S rDNA based conventional denaturing gradient gel electrophoresis (DGGE) analysis followed by next generation sequencing (NGS) on Illumina MiSeq platform. The core GBP of the tribal population of India was compared with the similar data on Hadza hunter-gatherers of Tanzania and urban Italians^21^, Mongolians (Mongolia)^22^, Malawians (Malawi)^7^, Amerindians (Venezuela)^7^ and Americans (USA metropolitan)^7^.

## Results

### DGGE-based analysis of GBP of the tribes of India

The GBP data were obtained from a total of 193 individuals (apparently healthy) across 15 ethnic groups of India distributed in four geographical locations namely Assam, Telangana, Sikkim and Manipur. Each ethnic group was distinct in their culture, tradition and dietary habits. The staple food of all the ethnic groups was rice with variation in consumption of vegetables, fish, meat, legumes, whole grains, fruits and tubers. The tribes from Manipur and Sikkim consume relatively higher quantity of fermented foods such as fermented bamboo shoot, fermented soy bean, fermented mustard seeds and leaves, along with some dried and smoked fish and meat in each servings. The Sikkim tribes consume more milk products in their diet compared to the others. Fecal samples from each ethnic group were collected from both male and female within the age group of 20–35 years, who had not taken antibiotics within three months prior to sample collection. The GBP of the volunteers were studied using DGGE analysis of PCR amplified V6-V8 region of bacterial 16S rDNA from the fecal metagenomic DNA. The DGGE profiles have been presented in the Fig. 2. Each of individuals had unique profiles irrespective of their ethnicity or geographies. However, some degree of similarity exists among the volunteers of each tribe and among the volunteers of one geographical location. Unweighted Pair Group Method with Arithmetic Mean (UPGMA) tree based on Dice co-efficient shows grouping of the individuals based upon their ethnicity (Fig. 2a–d). Multidimensional Scaling (MDS) plot of the DGGE data indicates the tendency of geography wise grouping of the subjects (Fig. 2e). Test of analysis of similarity (ANOSIM) indicated significant separation between the tribes of Telangana and Assam (R = 0.17, *p* < 0.01) and Manipur and Sikkim (R = 0.32, *p* < 0.01). When the DGGE data of the population was subjected to MDS analysis based on their racial origin, either Mongoloids or Proto-Australoids, some degree of separation of the Mongoloids from the Proto-Australoids was observed (R = 0.23, *p* < 0.01) (Fig. 2f). The Tea tribe of Assam though of Proto-Australoid origin had similar separation from both Proto-Australoid and Mongoloid groups (R = 0.17 & 0.18 respectively, *p* < 0.01).

### NGS-based analysis of GBP of the tribes of India

Amplicon sequencing on Illumina MiSeq platform covering V3-V4 region of bacterial 16S rDNA of the 75 fecal DNA samples representing 15 different tribes from the four geographies produced on an average of 380,827 high quality reads (average read length 449 nt) in each sample (Table S1). On an average 369,639 reads had significant homology with predicted rRNA sequences in the Ribosomal Database Project (RDP) resource. Slope of the rarefaction curves indicated sufficient coverage of bacterial diversity (Fig. S1). Overall, the GBP of the tribes of the four geographies had similar alpha (α), Simpson and Shannon diversity indices (Fig. S2). However, GBP of the tribes from Manipur had more Simpson diversity and less Shannon diversity compared to the tribes of Telangana (*p* < 0.05).

The phylum level distribution of bacteria in the gut of the volunteers from across the four geographies have been presented in the Fig. 3. The major bacterial phyla detected were *Firmicutes, Bacteroidetes* and *Actinobacteria.* The tribes of Manipur had significantly lower *Firmicutes* to *Bacteroidetes* ratio (F/B) in comparison to the tribes of Telangana and Assam (*p* = 0.014 & 0.040, respectively) (Fig. S3). The tribes from Sikkim contained significantly more *Actinobacteria* in comparison to the other tribes (*p* ≤ 0.005) (Fig. 3a–d).

The major bacterial families representing the GBP of the Indian population included in the study were *Prevotellaceae*, *Ruminococcaceae*, *Eubacteriaceae*, *Lachnospiraceae*, *Clostridiaceae*, *Veillonellaceae*, *Bacteroidaceae*, *Bifidobacteriaceae*, *Erysipelotrichaceae*, *Lactobacillaceae* and *Coriobacteriaceae* (Fig. 3e) of which *Bifidobacteriaceae*, *Lactobacillaceae*, *Veillonellaceae*, *Clostridiaceae* and *Eubacteriaceae* showed significant differences in their abundance across the population (*p* ≤ 0.05 Kruskal-Wallis H test). *Prevotella* (Phylum: *Firmicutes*) was the most dominant genus in all the tribes (Fig. 3f). On an average 40% of the gut bacteria belonged to the genus *Prevotella*. There were many unclassified genera belonging to the families *Ruminococcaceae, Erysipelotrichaceae* and the order *Clostridiales*. The bacterial genera accounting for significant variation across the tribes were found to be *Bifidobacterium*, *Gordonibacter*, *Slackia*, *Bacteroides*, *Odoribacter*, *Parabacteroides*, *Clostridium*, *Enterobacter*, *Escherichia*, *Klebsiella* and *Pantoea* (*p* ≤ 0.05, Kruskal-Wallis H test).

Principal Component Analysis (PCA) based on the NGS data at different taxonomic levels (phylum, class, order, family and genus level) were conducted (Fig. 4a–e). Distinct clustering of the subjects were observed based on their geographies at lower taxonomic levels (in increasing order) where the R value (ANOSIM) increased from 0.038 (phylum level; *p* = 0.089) to 0.357 (genus level; *p* = 0.0001) (Fig. 4a–e). The GBP of the tribes of Assam were more similar to the tribes from Telangana (R = 0.340; *p* = 0.0001) than the tribes of Sikkim (R = 0.508; *p* = 0.0001) and Manipur (R = 0.502; *p* = 0.0001). Heatmaps were generated based on the NGS data to see the distribution of bacterial taxa among the tribes belonging to different geographies (Fig. 4f–j). Similar pattern of grouping as in the case of PCA was observed in the heatmaps also.

### The core and unique gut bacteria of the Indian tribes

Among the 593 bacterial genera detected, bacteria which were present in at least 80% of the population with more than 0.1% relative abundance were considered as the core gut bacteria (Table 1). The core gut bacteria of the tribes of Telangana and Assam consisted of *Prevotella, Faecalibacterium, Eubacterium*, *Clostridium*, *Blautia, Collinsella*, *Ruminococcus* and *Roseburia.* Additionally, the bacterial genera *Bacteroides, Dialister* and *Veillonella* were also found to be core bacteria in the tribes of Manipur and *Bacteroides, Dialister, Bifidobacterium* and *Lactobacillus* in the tribes of Sikkim. The overall core gut bacteria of all these tribes of India consisted of *Prevotella*, *Faecalibacterium, Eubacterium*, *Clostridium*, *Blautia*, *Collinsella*, *Ruminococcus* and *Roseburia* which were present in the gut irrespective of their ethnicity, dietary habit and geographies.

The networks of co-occurring core genera were constructed based on their correlation matrix (Fig. 5). The tribal population from each geographic region had unique network of co-occurrence. In the tribes of Assam, *Prevotella* was found to be in negative correlation with *Faecalibacterium* and *Blautia* {*r* = −(0.433–0.756), *p* ≤ 0.031} while in positive correlation with *Collinsella* and *Veillonella* (*r* = 0.455–0.498, *p* < 0.022) (Fig. 5a). Among the core bacteria of the Telangana tribes, a negative correlation of *Prevotella* was observed with *Bacteroides, Faecalibacterium* and *Clostridium* {*r *= −(0.572–0.700), *p* ≤ 0.008} (Fig. 5b). In the tribes of Manipur, *Prevotella* was found to be in negative correlation with *Faecalibacterium* and *Roseburia* {*r* = –(0.565–0.647), *p* ≤ 0.028} (Fig. 5c). In the tribes of Sikkim, *Prevotella* was found to be in negative correlations with *Clostridium*, *Ruminococcus and Blautia* {*r  *= −(0.515–0.775),*p* ≤ 0.05}, while a positive correlation was observed between *Bifidobacterium* and *Lactobacillus* (*r* = 0.743, *p* = 0.002) (Fig. 5d). The tribes of Sikkim contained significantly less *Enterobacter* (*p* ≤ 0.01), *Klebsiella* (*p* ≤ 0.001) and *Pantoea* (*p* ≤ 0.003) in comparison to the tribes from Assam, Telangana and Manipur. Tribes of Assam had significantly more *Escherichia* (*p* ≤ 0.003) in comparison to the tribes from Telangana (*p* ≤ 0.03) and Sikkim (*p* ≤ 0.005). *Bacteroides,* one of the core gut bacteria of the tribes from Sikkim and Manipur was found to be in negative correlation with *Prevotella* {*r* = −(0.565–0.700), *p* ≤ 0.028} (Fig. 5c,d).

Other than the common core gut bacteria, there were also unique bacteria such as *Treponema* and *Gordonibacter* in the tribe Kolam, which were significantly more abundant in comparison to the other tribes (*p* < 0.05). Other such bacterial genera are *Phascolarctobacterium* in the tribe Tangkhul, *Selenomonas* in the tribe Nepali and *Pseudobutyrivibrio* and *Megasphaera* in the tribe Tai-Aiton (Table 1). Tribes of Sikkim had significantly more *Bifidobacterium* in their GBP compared to the tribes from Telangana, Manipur and Assam (*p* < 0.001), while tribes from Manipur had the least *Bifidobacterium*. Similarly, the abundance of *Lactobacillus* was higher in the tribes of Sikkim (*p* < 0.05).

### Comparison of GBP of the Indian tribes with the worldwide data

A comparison of GBP of the Indian tribes was performed with rural and urban communities of the world using data obtained from the MG-RAST server (Table S2) (Fig. 6). Data on GBP from earlier studies on Hadza gatherers (Tanzania), rural Malawians, rural Amerindians (Venezuela), Mongolians (Mongolia) and urban population from America and Italy were obtained (Fig. 6a). A PCA plot based on the data of genus level abundance showed distinct groups where Mongolians grouped closer to the Indians (R = 0.339; *p* ≤ 0.0001) compared to the others (R ≥ 0.412) (Fig. 6b). A heatmap analysis showed two distinct major clusters, in which Hadza, Italian and Americans separated from the rest of the groups (Fig. 6c). In the other major cluster, Malawians and Amerindians separated from the Indian tribes. Mongolian grouped together with the Indians and showed close similarity with the tribes of Mongolian origin (Nepali and Tai-Phake). *Prevotella* was the most dominant genus in the tribes of India along with the Mongolian, Amerindian and Malawian groups. While in the Hadza, Italian and American populations, *Faecalibacterium* was found to be dominated which clustered them separately from the *Prevotella* dominated group (Fig. 6c).

In the network of co-occurring genera of the core gut bacteria of the Indian tribal population, *Prevotella* was found to be negatively correlated with *Bacteroides*, *Faecalibacterium*, *Blautia*, *Clostridium* and *Ruminococcus* {r *=* −(0.336–0.407), *p* ≤ 0.003). While *Blautia* was found to be in positive correlation with *Faecalibacterium*, *Eubacterium*, *Clostridium* and *Roseburia* (r = 0.235–0.273, *p* < 0.037) (Fig. 7a). A comparison of the core gut bacteria of the Indian tribes with the representative population of the world led to the identification of core gut bacteria across the globe which consists of *Faecalibacterium*, *Eubacterium*, *Blautia*, *Clostridium, Ruminococcus* and *Roseburia* (Table S3). In the network of co-occurrence of core bacterial genera of the world population, except *Roseburia* all the bacteria were found to be in positive correlation with each other (r = 0.201–0.540, *p* < 0.012) (Fig. 7b).

## Discussion

The Mongoloid and Proto-Australoid tribes of India under study are yet to be touched by modern lifestyle who still rely on traditional agriculture, fishing, livestock farming and traditional medicinal practices and have their own dietary habits. Owing to their strong social cohesiveness, a tribal population forms a considerable homogeneous group to conduct such studies. However, the ongoing rapid industrial and economic development of India may alter their lifestyle in the near future. Taking advantage of these, this study on gut bacterial diversity across and within geographies and racial origins was undertaken. The result has for the first time, revealed the GBP of the tribal population of India. Analysis of GBP by classical DGGE technique coupled with NGS analysis has indicated a complex interaction among the factors, environment, diet and genetic determinants involved in shaping GBP of an individual.

The DGGE based analysis indicated the effect of geography and racial origin on GBP. MDS plot of the DGGE data based on the racial origin could also separate the individuals indicating the role of genetic determinants. The Tea tribe (Santhal) of Assam of Proto-Australoid origin who originally migrated from Jharkhand (over 100 years ago) grouped separately from the other Proto-Australoid tribes of Telangana and Mongoloid tribes of Assam indicating role of environment and genetic determinants on GBP. In the NGS analysis, however, the role of racial origin was not prominent. It could be because, the DGGE technique, though detected only a narrow window of diversity, yet it captured the strain level differences in that window of the GBP. On the other hand, the PCA and heatmap analysis of the NGS data was based on the % abundance up to genus level and thus, it was unable to account the differences in the various oligotypes present under each genera which may have relation with the genetic factors^23^.

Overall the NGS data indicated the dominant effect of geographies and diet on GBP of the Indian tribes. Both PCA and heatmap analysis showed that the tribes from Assam and Telangana had more similar GBP than from Sikkim and Manipur. The core GBP (defined by bacteria with >0.1% abundance in ≥80% of the population) was very similar between the tribes of Assam and Telangana despite their racial difference. The diet of the Indian tribes, in general has been rich in carbohydrates and dietary fibers which include rice, vegetables, legumes, whole grains, fruits, tubers etc. However, the tribes from Manipur and Sikkim consume more boiled vegetables and fermented foods. The tribes of Sikkim also consume more milk products in their diet compared to others. The F/B was significantly lower in the tribes of Manipur compared to the tribes from the other geographies. Previous studies on African and European groups indicated lower F/B due to plant fiber rich diet^15^. In contrast, a study on the Mongolian tribe who consumed more red meat, fermented dairy products and liquor showed low F/B^15,22^. Therefore, dietary habits may not determine the F/B.

Tribes from Sikkim had more *Actinobacteria* compared to the tribes from the other geographies. The phylum *Actinobacteria*, mainly represented by *Bifidobacteria* has been reported to be involved in protection against pathogens and maintenance of immune system and exertion of nutritional effects to the intestinal cells and the host^24^,^25^,^26^. The genera, *Bifidobacterium* and *Lactobacillus* were significantly more abundant in the tribes Lepcha, Bhutia and Nepali of Sikkim which may be due to their high intake of milk products (including churpi and curd) and fermented products containing *Bifidobacterium* and *Lactobacillus*^27^,^28^. Previous reports have also suggested that several food borne microbes, particularly dairy associated microbes can survive the transit through the digestive system^9^. *Bifidobacterium* strains found in the human gastrointestinal tract also exert antimicrobial activity, thus they participate in the “barrier effect” produced by the indigenous microbiota^29^,^30^. Higher altitude may also be another factor influencing the gut microbiota composition of the Sikkim tribes^31^.

Overall, the diet of the Indian tribes is rich in carbohydrates and as a result their GBP is also enriched in carbohydrate-metabolizing bacteria of the family *Prevotellaceae*^32^*. Ruminococcaceae*, *Lachnospiraceae* and *Eubacteriaceae* family members are involved in metabolism of carbohydrate into butyrate {a short-chain fatty acid (SCFA)} and gas^33^,^34^. SCFAs are important sources of energy for colonic epithelial cells and may enhance epithelial barrier integrity and modulate the gastrointestinal immune response^35^. In the Indian tribes, dominance of the genus *Prevotella* belonging to the *Prevotellaceae* family indicates the occurrence of Enterotype 2 as proposed by Arumugam *et al.*^36^. Similar study under the Asian Microbiome Project reported dominance of *Prevotella* in Indonesia and Khon Kaen in Thailand^37^. *Prevotella* was also found to dominate the GBP of the Mongolian, Amerindian and Malawian populations which clustered closely with Indian tribes which could be due to their carbohydrate rich diet^38^. As in the case of Yanomami, the uncontacted Amerindians, the GBP of the Indian tribes were composed of high *Prevotella* and low *Bacteroides* which may be due to their traditional lifestyle untouched by modernisation^39^. Higher abundance of *Treponema* in the Kolam and Karbi tribes could be due to their consumption of plant tubers as earlier reported in the Hadza group^21^.

Variation of GBP was found to exist between the tribes within a geographic region, although the differences were more prominent across geographies. Earlier reports indicated the differences in the GBP between individuals living in different countries (USA compared to Malawian and Amerindian)^7^. While GBP vary among individuals within a community or among the communities, the metabolic processes necessary for normal functioning of human gut may be conserved and a functional core microbiota appears to exist. Previous report from the Metagenomics of the Human Intestinal Tract (MetaHIT) project indicated 32 core bacterial species in more than 80% of the European population that belonged to the genera, *Faecalibacterium*, *Roseburia*, *Bacteroides*, *Dorea*, *Clostridium*, *Eubacterium*, *Coprococcus*, *Ruminococcus*, *Alistipes*, *Collinsella*, *Parabacteroides* and *Bifidobacterium*^4^. In this study, *Faecalibacterium*, *Eubacterium*, *Clostridium*, *Blautia*, *Ruminococcus* and *Roseburia* were found to be the core gut bacterial genera across the representative populations of the world (both rural and urban). *Faecalibacterium*, *Eubacterium*, *Clostridium*, *Ruminococcus* and *Roseburia* are important carbohydrate fermenting bacteria that are mostly involved in starch fermentation with the production of butyrate^40^. *Blautia* was reported to be host specific and its host preference is controlled by the host physiology rather than the dietary habit^23^. Previous studies reported that phylogenetically similar bacterial species tend to appear in the same individual, whereas they should actually compete with each other due to their overlapping functional roles or for habitats^41^. Clustering of the individuals of the Indian tribes based on their GBP at increasing order towards lower taxonomic levels also hints towards functional redundancy of gut microbiota.

Thus this study has for the first time revealed the GBP of the Mongoloid and Proto-Australoid tribes of India and indicated the effect of a complex interaction among the factors, environment, diet and genetic determinants involved in shaping the GBP of an individual. A comparison with the representative worldwide data on GBP, a core set of bacterial genera has been identified. Further research will be carried out to understand the role of the GBP on health of the individuals of these tribal populations.

## Methods

### Recruitment of volunteers

**Figure 1:**
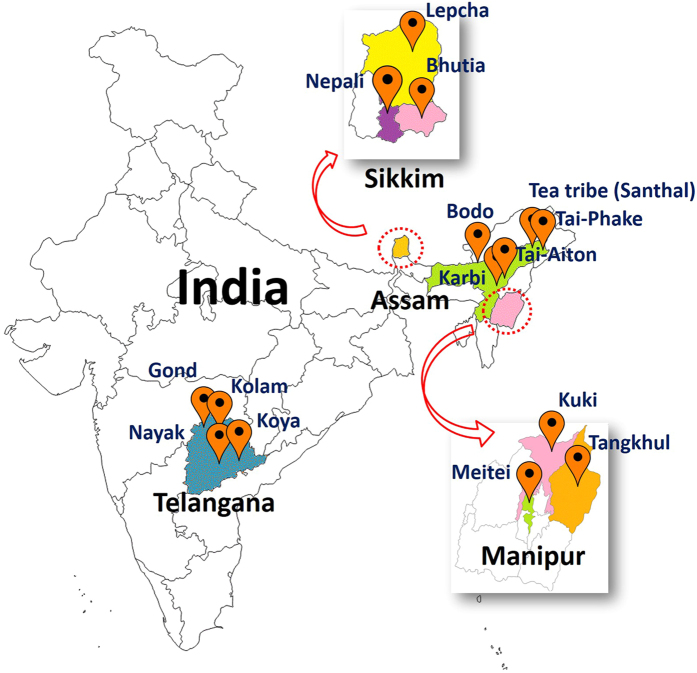
Sampling sites in India. Volunteers were recruited from 15 tribal populations from 4 geographic regions of India. (Map has been adapted from http://d-maps.com/carte. php/num_car=24853&lang=en).

**Figure 2:**
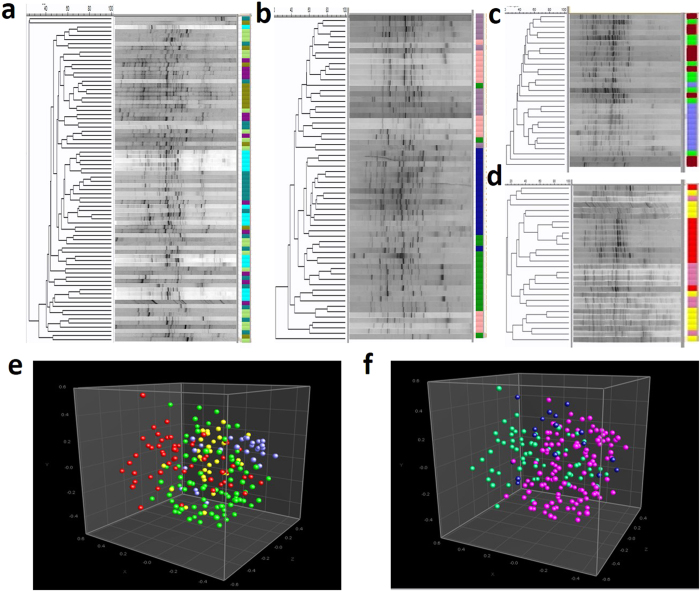
Gut bacterial profile of the Indian tribes. (**a**) DGGE gel images of PCR-amplified 16S rRNA gene of fecal DNA of Assam (Bodo, ocean blue; Karbi, moss green; Tai-Aiton, cyan; Tai-Phake, purple and Santhal, yellow green), (**b**) Telangana (Kolam, orchid color; Nayak, lavender; Koya, dark green; and Gond, dark blue), (**c**) Manipur (Kuki, brown; Meitei, green and Tangkhul, sky blue,), and (**d**) Sikkim (Bhutia, red; Lepcha, yellow and Nepali, pink). Each lane indicates the bacterial profile of one volunteer and each band represents a bacterium. The UPGMA tree in the left hand side indicates closeness between bacterial profiles of the volunteers (Each tribe color coded). Multidimensional scaling (MDS) plot of the DGGE profiles indicates the distribution of the volunteers with respect to their (**e**) Geographic region, (Telangana, red balls; Assam, green balls; Sikkim, navy blue and Manipur, yellow balls) and (**f**) Racial origin, (Mongoloids, dark pink balls; Proto-Australoids, dark green balls and Tea tribe (Santhal) from Assam in dark blue balls).

**Figure 3:**
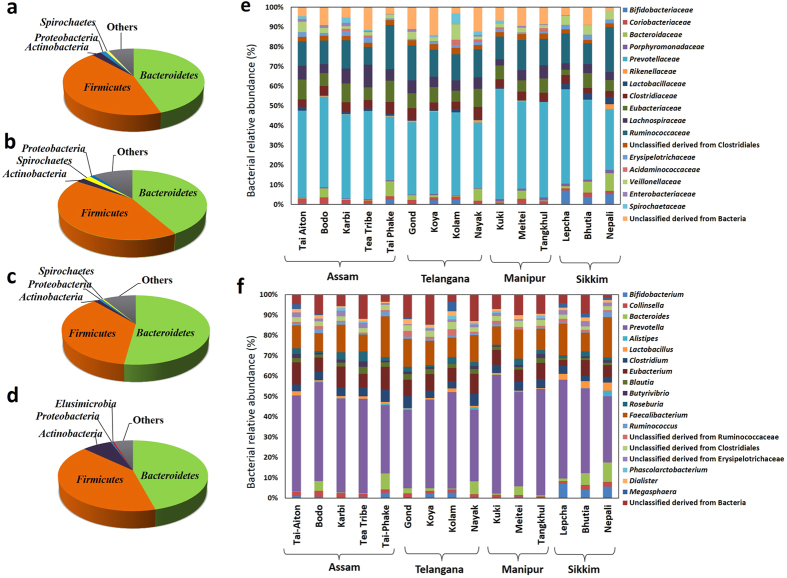
Relative abundance of the bacterial groups representing the gut bacteria of the tribes of India. Pie charts showing phylum level distribution of bacterial relative abundance (%) in the gut of the tribes of (**a**) Assam, (**b**) Telangana, (**c**) Manipur, and (**d**) Sikkim. Relative abundance of gut bacteria at (**e**) Family and (**f**) genus level among the tribes of India of different geographies. The bacterial families and genera represented in the histogram shows relative abundance ≥1% in at least 10% of the subjects.

**Figure 4:**
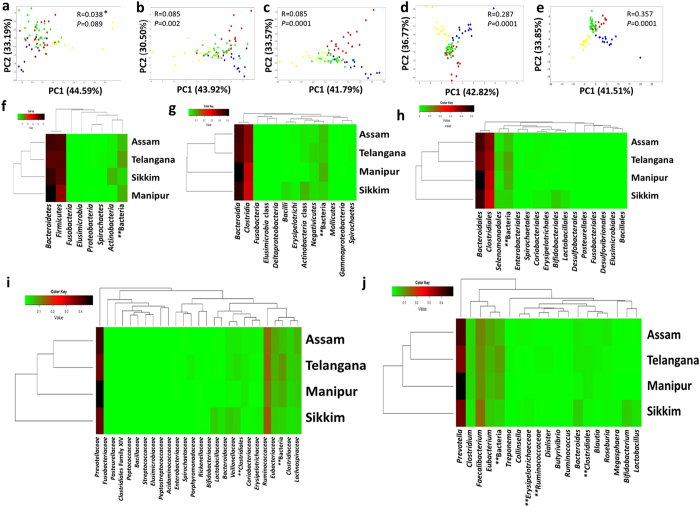
Comparison of gut bacterial profile of the four geographies at different taxonomic levels. PCA analysis was carried out and the corresponding heatmaps were constructed with the abundant gut bacteria at (**a**,**f**) phylum, (**b**,**g**) class, (**c**,**h**) order, (**d**,**i**) family and (**e**,**j**) genus level (Telangana, red balls; Assam, green balls; Sikkim, blue balls and Manipur, yellow balls). {*Analysis of similarity (ANOSIM), **Unclassified bacteria}.

**Figure 5:**
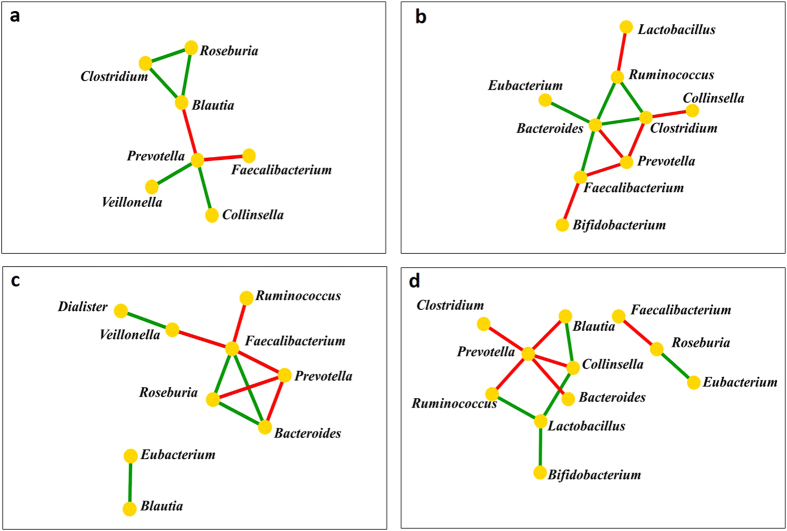
Network of co-occurring genera of the core gut bacteria of the fifteen tribes from different geographies. Network of co-occurring genera were constructed in Cytoscape using correlation data of core gut bacteria of the tribes of (**a**) Assam, (**b**) Telangana, (**c**) Manipur and (**d**) Sikkim. The nodes represent bacterial genera and the edges represent the type of correlation (Green: positive; red: negative).

**Figure 6:**
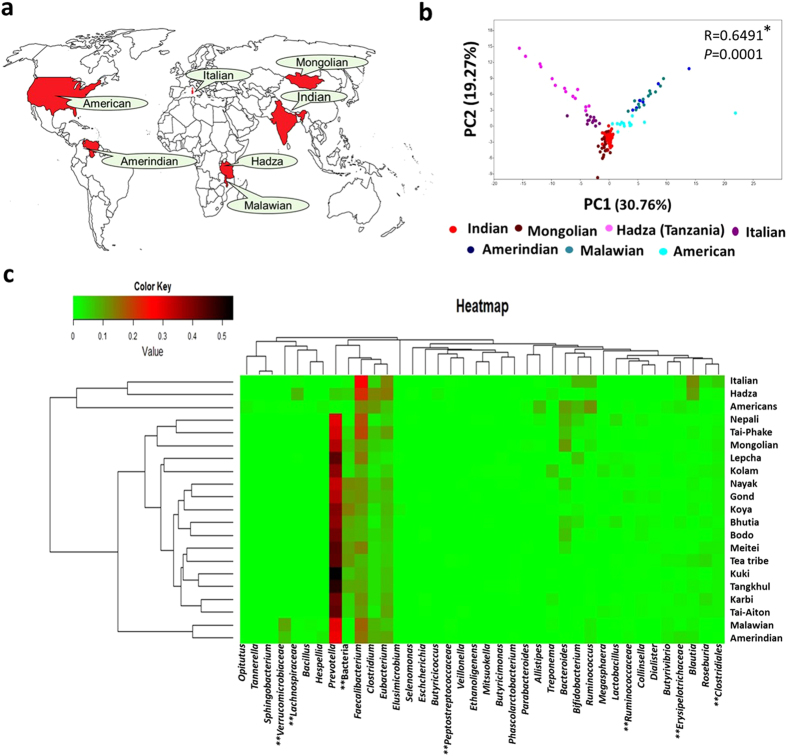
Comparison of gut bacterial profile of Indian tribes with the rest of the world. (**a**) The gut bacterial profile of the Indian tribes were compared with the hunter gatherer group- Hadza, rural Malawians, Amerindians and Mongolians, also with urban populations from Italy and USA. (**b**) A PCA analysis was carried out based on the bacterial relative abundance (%) data. (**c**) A heatmap was constructed based on the gut bacterial profile of the 15 Indian tribes along with the Mongolian, Italian, Hadza, Amerindians, Malawian and Americans. {*Analysis of similarity (ANOSIM), **Unclassified bacteria} (Map has been adapted from http://d-maps.com/carte.php?num_car=13181&lang=en).

**Figure 7:**
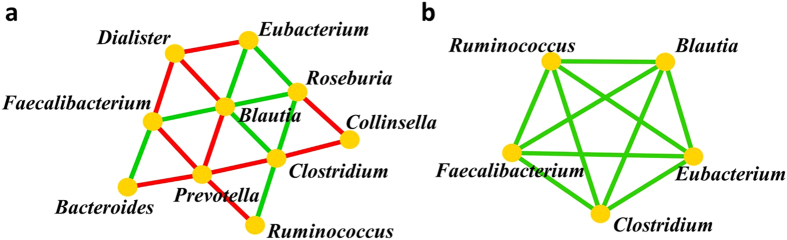
Network of co-occurring genera of the core gut bacteria of the Indian tribes and representative population of the world. Network of co-occurring genera were constructed in Cytoscape using correlation data of core gut bacteria of (**a**) Indian tribes and (**b**) the representative population of the world. The nodes represent bacterial genera and the edges the type of correlation (Green: positive; red: negative).

A total of 193 healthy volunteers belonging to 15 different ethnic groups from four different states of India were recruited for the current study (Fig. 1). All the ethnic groups live in the rural areas away from the modern lifestyles and entirely rely on agriculture for livelihood. Among the 193 volunteers, (i) 78 were from the Bodo, Karbi, Tai-Phake, Tai-Aiton and Tea Tribe (Santhal) of Assam, (ii) 27 were from the Lepcha, Bhutia and Nepali tribes of Sikkim, (iii) 30 were from the Meitei, Kuki and Tangkhul tribes of Manipur and (iv) 58 were from the Nayak, Koya, Kolam and Gond tribes of Telangana. Except the Tea Tribe (Santhal), all the tribes of Assam, Manipur and Sikkim are of Mongoloid race. While the Tea Tribe (Santhal) of Assam and all the tribes of Telangana are of Proto-Australoid race. Healthy individuals without any gastrointestinal disorder, who did not take any antibiotics for at least three months before sampling time and who were in the age group of 20–35 years were included (both male and female). This study was approved by Human Ethical Committee, Institute of Advanced Study in Science & Technology (IASST), Guwahati, India. All the experiments were performed in accordance with relevant guidelines and regulations. Written informed consents were taken from the volunteers along with a standard questionnaire.

### Sample collection and preservation

Fecal samples were collected from the volunteers in RNAlater™ (Cat. no. 76104, QIAGEN, Germany) solution in sterile stool collection tubes and stored at −80˚C immediately after transportation to the laboratory^42^. Details of the dietary habit, age and physical status of the volunteers were recorded (Table S4). Volunteers were categorized into 4 groups based on geographical location and into 15 groups based on their tribe.

### DNA extraction, PCR amplification and denaturing gradient gel electrophoresis (DGGE) of V6-V8 region of bacterial 16S rDNA

In this study we compared the GBP of the 193 individuals representing 15 different tribes of India using DGGE analysis. Metagenomic DNA was extracted from the fecal samples using QIAGEN DNA Stool Mini-Kit (QIAGEN, Hilden, Germany)^43^. PCR amplification of the V6-V8 region of the bacterial 16S rDNA was carried out using the primer pair, U968GC (5′CGCCCGCCGCGCGCGGCGGGGCGGGGCGGGGCACGGGGGGAACGCGAAGAACCTTAC3′) and L1401 (5′CGGTGTGTACAAGACCC3′)^44^. PCR reaction was performed in a 25μl volume in a thermal cycler (Mastercycler Nexus gradient, Eppendorf, Germany). Each PCR reaction contained a final concentration of 1x standard buffer, 1.75 mM of MgCl_2,_ 200 μM of dNTPs, 0.2 μM of each primer, 1 U of Taq DNA polymerase (Sigma Aldrich, USA) and 25 ng of template DNA. PCR conditions were, initial denaturation at 94 °C for 5 min. followed by 35 cycles of denaturation at 94 °C for 30 sec., annealing at 55 °C for 30 sec., extension at 72 °C for 30 sec. and a final extension at 72 °C for 7 min. PCR products were separated in a 2% agarose gel along with 50 bp mass ladder for size and mass calculation of PCR product and visualized under BioDoc-It Imaging System (UVP, USA). Band quantification was performed using ImageJ software comparing with the mass ladder^45^.

The DGGE was performed in a 9% acrylamide: bisacrylamide (37.5:1) gel using 35% to 60% denaturant gradient using Ingeny PhorU-2 DGGE system (Ingeny International BV, Goes, Netherlands). A 100% denaturant contained 7 M urea (Promega, USA) and 40% deionized formamide (Sigma Aldrich, USA). A 6% stacking gel was poured at the top of the denaturing gel and 600 ng of the PCR product was loaded for each sample. A PCR amplified product of *E. coli* DNA was used as control sample. A reference sample was developed by mixing few samples having most of the representative bands. The electrophoresis was carried out at 70 V for 17 hours at 60 °C in 1X TAE buffer. The DGGE profile were analyzed in Gel Compar II version 6.6 (Applied Maths, Belgium) using a mix of DNA profiles as a marker. Due to inherent limitations of DGGE technique including its inability to detect a larger portion of gut bacterial diversity, NGS analysis was carried out.

### Next Generation Sequencing (NGS) on Illumina MiSeq platform

Metagenomic DNA from 75 samples representing 15 tribal groups which included 5 random samples from each tribe, including both male and female were further subjected to Next Generation Sequencing (NGS) with Xcelris Genomics (Ahmedabad, India). Bacterial diversity in the samples were analyzed using V3-V4 region of 16S rDNA amplicon sequencing on Illumina MiSeq platform. Quantification of the DNA was performed using Qubit dsDNA BR Assay kit (Thermo Fisher Scientific, USA). A 2 × 300 bp of MiSeq amplicon library was prepared using the Nextera XT Index kit (Illumina Inc., USA) as per the 16S metagenomic sequencing library preparation protocol (Part # 15044223 Rev. B). The primer pair, V3-forward (5′CCTACGGGNGGCWGCAG3′) and V4-Reverse (5′GACTACHVGGGTATCTAATCC3′) were designed and synthesized for the amplification of V3–V4 region of 16S rDNA gene of Eubacteria and Archaea. Amplicons were then ligated with Illumina adaptors and were amplified by using i5 and i7 primers that add multiplexing index sequences as well as common adapters required for cluster generation (P5 and P7) as per the standard Illumina protocol. The amplicon libraries were purified using 1X Ampure XP beads and checked on Agilent DNA 1000 chip on bioanalyzer 2100 and quantified on fluorometer by Qubit dsDNA HS Assay kit (Life Technologies, India). After obtaining the Qubit concentration for the library and the mean peak size (~600 to ~630 bp) from bioanalyser profile, 600 μl of 10 ρM pooled libraries (spiked with 5% 12.5 ρM PhiX Control) was loaded into MiSeq reagent cartridge for cluster generation. Cluster generation was carried out by hybridization of template molecules onto the oligonucleotide-coated surface of the flow cell. Immobilized template copies were amplified by bridge amplification to generate clonal clusters. The kit reagents were used in binding of samples to complementary adaptor oligos on paired end flow cell. The adaptors were designed to allow selective cleavage of the forward strand after resynthesis of the reverse strand during sequencing. The copied reverse strand was then used to sequence from the opposite end of the fragment. Barcode and sequencing primers were trimmed from sequences. The trimmed sequences in FASTQ file was then uploaded to Metagenomic RAST server (MG-RAST)^46^, which was then preprocessed to remove the low quality regions of FASTQ data using SolexaQA. Sequences with an average Phred score lower than 25, containing ambiguous bases, homopolymer run exceeds 6, having mismatches in primers, or sequence length shorter than 100 bp were removed. Details of the samples with individual MG-RAST ids have been presented in the Table S1. The analysis was performed in the MG-RAST server within Ribosomal Database Project (RDP) and taxonomic assignment was carried out with 97% homology match. Bacterial abundance data at phylum, class, order, family and genus levels were downloaded from the MG-RAST server. Similarly, the bacterial abundance data (genus level) of previously published work on American, Malawian, Amerindian, Hadza, Italian and Mongolian populations of the same age group were collected from the MG-RAST server (Table S2). Bacterial genera with abundance value >0.1% in atleast 80% of the individuals of a population was considered as a core member of GBP.

### Statistical Analysis

Rarefaction curve for each individual sample was generated in the MG-RAST server. To analyze further the diversity of the gut ecosystem, alpha (α), Shannon and Simpson diversity indices were calculated as described earlier^47^ and to test the significant differences, one-way ANOVA was performed within Statistical Package for Social sciences (SPSS) with Post Hoc pairwise Least Significance Difference (LSD) comparison (IBM SPSS 20, SPSS Inc, Chicago, IL). The non-normally distributed data on α-diversity were transformed by Johnson transformation within Minitab (Minitab 17, State College, Pennsylvania, England). Percentage abundance data at different taxonomic levels were further used to analyze the variation in the GBP. Principal component analysis (PCA) was performed in PAleontological STatistics (PAST) (version 3.04) software using the bacterial relative abundance (%) data after normalization (x-mean/standard deviation). Significant test for the clustering of the samples in the PCA analysis was carried out in PAST using one-way analysis of similarities (ANOSIM). Data of the DGGE analysis from the GelCompar II software were exported as band matching table and one-way ANOSIM analysis performed within PAST. Heatmaps were constructed within R-statistical tool using rcolorbrewer, vegan, gplot and heatplus packages. In the heatmap, the bacterial relative abundance of more than 0.01% (phylum, class, order, family) and for genus level analysis bacterial relative abundance of more than 0.1% were considered. In the hierarchical tree of the heatmaps, the X-axis indicated the similarity in abundance profile of the bacterial taxa and the Y-axis indicated similarity among the GBP of different geographies or tribes based on Bray-Curtis similarity matrix. Kruskal-Wallis H test was performed within SPSS to find out the bacteria which were significantly different among the tribes. Comparison of relative abundance data of selected bacterial genera across the tribes was performed using Mann-Whitney *U* test within SPSS (IBM SPSS 20, SPSS Inc, Chicago, IL). To construct the network of co-occurrence of the core gut bacteria of different geographies bivariate correlation analysis was performed {Pearson correlation (parametric) or Spearman correlation (non- parametric) (IBM SPSS, statistics 20)} and the network was generated using Cytoscape^48^ (version 3.2.1). Networks were visualized using prefuse force directed layout where the nodes represent the bacterial genera and the edges represent the correlation (negative-red; positive-green).

## Additional Information

**How to cite this article**: Dehingia, M. *et al.* Gut bacterial diversity of the tribes of India and comparison with the worldwide data. *Sci. Rep.* **5**, 18563; doi: 10.1038/srep18563 (2015).

## Supplementary Material

Supplementary Information

## Figures and Tables

**Table 1:** Core gut bacterial genera and their relative abundance in different tribes of Telangana, Manipur, Sikkim and Assam.

Core bacterial genera	Assam						Telangana			Manipur			Sikkim		
	Tai-Phake	Tea tribe	Tai-Aiton	Bodo	Karbi	Gond	Koya	Nayak	Kolam	Tangkhul	Kuki	Meitei	Nepali	Bhutia	Lepcha
*Prevotella*	31.69	43.8	43.14	41.42	42.12	35	39.69	33.18	38.94	49.01	53.46	44.07	30.42	38.87	43.44
*Faecalibacterium*	18.73	7.92	10.25	8.41	11.27	12.2	10.47	12.02	8.15	9.83	8.76	13.65	17.96	8.53	14.04
*Eubacterium*	10.37	6.04	9.79	6.74	8.8	7.04	7.57	8.68	5.48	7.43	7.12	5.57	5.09	7.11	2.87
*Clostridium*	6.67	4.04	3.10	4.38	4.15	6.17	3.28	5.30	3.20	4.17	4.00	4.16	2.5	2.45	3.86
*Blautia*	2.13	3.38	1.92	1.59	1.91	2.12	2.24	2.34	1.90	2.18	1.77	1.09	1.12	1.01	1.38
*Collinsella*	1.41	1.31	2.05	2.52	1.93	1.70	1.19	1.41	1.24	0.60	1.312	0.79	2.43	2.22	1.22
*Ruminococcus*	2.38	0.41	0.61	1.9	1.25	1.01	0.84	0.63	0.93	0.56	1.06	0.25	2.23	0.48	0.54
*Roseburia*	1.52	4.36	2.65	0.97	3.92	1.71	0.91	1.49	2.28	1.81	0.63	2.83	1.25	2.25	0.92
*Bacteroides*	6.96	0.25		7.24	0.39	2.66	1.19	5.60		0.52	0.43	4.03	9.05	5.54	0.99
*Dialister*			1.54	1.53	1.11	2.57	1.4		1.97	0.82	0.96	1.8	1.14	1.77	1.16
*Butyrivibrio*		2.23	1.76				0.82	1.15		1.98		1.13	0.92	0.78	
*Bifidobacterium*	2.31		0.81			0.30	2.31	0.49			0.29		5.33	3.81	2.64
*Lactobacillus*	0.95		1.73		1.10	0.25	0.60		1.62		0.72	0.20	3.77	3.36	6.68
*Butyricicoccus*				0.23		0.25	0.22		0.32						
*Butyricimonas*								0.55	1.41	0.90	0.36				
*Veillonella*		0.58	1.12	0.34	0.17			0.55		0.23	0.33	0.14		0.39	
*Ethanoligenens*				0.36		0.85			1.04						
*Parabacteroides*				0.15		0.57				0.16		0.54			
*Mitsuokella*						0.42							0.75		
*Alistipes*			0.40			0.91						0.46			
*Treponema*									6.09						
*Desulfohalobium*				0.15			0.16								0.18
*Streptococcus*		0.19							0.22						0.14
*Gordonibacter*									0.20						
*Erysipelothrix*									0.46				0.21		0.23
*Escherichia*			0.53								0.31	0.34			
*Phascolarctobacterium*										1.31					
*Selenomonas*													0.92		
*Pseudobutyrivibrio*			0.19												
*Megasphaera*			2.26												
