# Supplementary material for: Gut bacterial diversity of the tribes of India and comparison with the worldwide data
Source: Sci Rep. 2015 Dec 22;5:18563. doi: 10.1038/srep18563 (PMC4686986; doi:10.1038/srep18563)
Supplement: Supplementary Information [file srep18563-s1.doc]

**Supplementary Information**

**Gut bacterial diversity of the tribes of India and comparison with the worldwide data**

Madhusmita Dehingia**^1^**, Thangjam Kanchal devi^2^, Narayan C. Talukdar^1,2^, Rupjyoti Talukdar^3^, Nageshwar Reddy^3^, Sharmila S. Mande^4^, Manab Deka^5^ and Mojibur R. Khan^1*^

**^1^**Molecular Biology and Microbial Biotechnology Laboratory, Life Science Division, Institute of Advanced Study in Science and Technology (IASST), An autonomous institute under Department of Science and Technology (Govt. of India), Paschim Boragaon, Garchuk, Guwahati - 781035, Assam, India.

^2^Institute of Bioresources & Sustainable Development (IBSD), An autonomous institute under Department of Biotechnology (Govt. of India), Takyelpat, Imphal - 795001, Manipur, India.

^3^Asian Healthcare Foundation/Asian Institute of Gastroenterology (AIG), 6-3-661, Somajiguda, Hyderabad-082, Telangana, India.

^4^Head of Bio-Sciences R&D, TCS Innovation Labs, Tata Consultancy Services Ltd. 54-B, Hadapsar Industrial Estate, Pune -411013, Maharastra, India.^5^Head of Department of Applied Sciences, Gauhati University, Guwahati-781014, Assam, India.

*Corresponding author email: mojibur.khan@gmail.com

Phone: +91-361-2273058         Fax: +91-361-227306

**Supplementary methods**

**Recruitment of volunteers**

The objective of this research was to study the gut bacterial profile (GBP) of Proto-Australoid and Mongoloid tribes, untouched by modern lifestyle living in South and North-East of India for understanding the role of ethnicity and geography on GBP. Under these two major racial groups many other ethnic groups who are culturally distinct, follow different dietary habits and living in different geographies were included. The South (Telangana) and North- Eastern states of India are the major habitats of the tribal populations of Proto-Australoid and Mongoloid origins, respectively. The Tea-tribe (Santhal) of Assam (North-East India) are of Proto-Australoid origin migrated 100 years ago from Jharkhand to work in the tea gardens of Assam was also included for better understanding of the effect of ethnicity and environment. The populations included in this study are yet be touched by modern lifestyle.

The North-Eastern states Assam, Sikkim and Manipur were included for this study. Geographical, ecological and ethnical diversity within the states makes the North-East quite different from other parts of the subcontinent. Sikkim is geographically different from Assam and Manipur due to its location in the Himalayas and the climate ranges from subtropical to high Alpines. Almost the entire state is hilly with elevation ranging from 920 ft to 28,169 ft and the average annual temperature for most of the Sikkim is around 18 °C. Thus we have selected the three major tribes from Sikkim which were Bhutia, Nepali and Lepcha (hilly tribes). While Manipur is a rich valley surrounded by hills and lake at an elevation of 2,592 ft above the sea level and thus it has both hilly (Tangkhul and Kuki) and plain tribes (Meitei) occupying the hilly and the valley areas which were included in the study. Maximum temperature in Manipur rises to 32 ̊C while winter temperature falls below zero in some places of Manipur. Assam has three different geographical regions, the Northern Himalayas (Eastern Hills), Northern Plain (Brahmaputra valley) and Deccan Plateau (Karbi Anglong). The Bodo tribe was selected from the lower Assam region and Tai Phake and Tea-tribe from the upper Assam region of the Brahmaputra valley. The Tai-Aiton and Karbi tribes were selected from the middle Assam region. Other than Karbi, all other tribes are from the plains. Assam has a sub-tropical climate (summer maximum 35-38 ̊C and winter minimum 6-8 ̊C) with heavy rainfall and high humidity. On the otherhand, the state Telangana from South India has its own distinctive culture and identity. Telangana is situated on the [Deccan Plateau](https://en.wikipedia.org/wiki/Deccan_Plateau), in the central stretch of the eastern sea board of the Indian Peninsula. Telangana is a semi-arid area and has a predominantly hot and dry climate with average maximum temperature 42 °C and winter is mild and dry with average temperature 22–23 °C. Four tribes were included from Telangana, viz. Kolam and Gond tribe from Adilabad district and Nayak and Koya from Khammam district.

Healthy individuals (both male and female) without any gastrointestinal disorder, in the age group of 20 - 35 years, without any antibiotics for three months prior to sample collection were included. A total of 193 volunteers belonging to 15 different ethnic groups from four different states of India were recruited. Among the 193 volunteers, 20 Bodo, 12 Karbi, 12 Tai-Phake, 16 Tai-Aiton and 18 Tea Tribe representing Assam, 10 Lepcha, 10 Bhutia and 8 Nepali representing tribes from Sikkim, 10 Meitei*,* 10 Kuki and 10 Tangkhul representing tribes of Manipur and 15 Nayak*,* 16 Koya*,* 11 Kolam and 15 Gond representing tribes of Telangana were included. Details about the volunteers are presented in the Supplementary Table S4.

**Table S1** Details of the Next Generation Sequencing analysis.

| **Tribe name** | **Sample id** | **MG-RAST id** | **MG-RAST code** | **Base pair count** | **Total reads** | **Quality reads** | **α-diversity** |
| --- | --- | --- | --- | --- | --- | --- | --- |
| Bodo | BM15 | 4556487.3 | MK1 | 16,55,73,928 | 366,124 | 364,218 | 69.485 |
| Bodo | BM20 | 4553197.3 | MK2 | 25,98,28,200 | 575,188 | 574,738 | 15.561 |
| Bodo | BF06 | 4553198.3 | MK3 | 26,30,24,516 | 579,568 | 579,007 | 18.992 |
| Bodo | BF04 | 4565522.3 | MK4_1 | 32,14,03,341 | 739,921 | 738,007 | 25.629 |
| Bodo | BF05 | 4553287.3 | MK5 | 28,22,59,108 | 625,102 | 624,581 | 36.213 |
| Tai-Aiton | AF12 | 4553288.3 | MK6 | 25,23,82,874 | 558,074 | 557,519 | 34.452 |
| Tai-Aiton | AF15 | 4553289.3 | MK7 | 23,64,50,853 | 523,294 | 522,744 | 22.2 |
| Tai-Aiton | AM04 | 4553290.3 | MK8 | 21,30,36,557 | 472,759 | 472,099 | 52.28 |
| Tai-Aiton | AM05 | 4553291.3 | MK9 | 16,53,13,831 | 364,824 | 362,163 | 26.572 |
| Tai-Aiton | AM03 | 4553292.3 | MK10 | 22,97,62,517 | 508,829 | 508,428 | 23.801 |
| Tea tribe | SM01 | 4553363.3 | MK11 | 19,06,47,196 | 422,072 | 420,921 | 11.624 |
| Tea tribe | SM03 | 4565521.3 | MK12_1 | 27,62,04,583 | 639,992 | 638,902 | 32.541 |
| Tea tribe | SM07 | 4553355.3 | MK13 | 19,96,02,747 | 442,218 | 441,893 | 51.01 |
| Tea tribe | SF13 | 4553356.3 | MK14 | 18,02,91,322 | 396,553 | 396,073 | 22.256 |
| Tea tribe | SF14 | 4553357.3 | MK15 | 20,24,60,066 | 451,622 | 451,249 | 28.492 |
| Karbi | KaM14 | 4553328.3 | MK16 | 17,54,40,332 | 388,539 | 387,999 | 39.313 |
| Karbi | KaF15 | 4553329.3 | MK17 | 20,04,04,586 | 443,906 | 443,310 | 42.595 |
| Karbi | KaM18 | 4553330.3 | MK18 | 25,61,99,688 | 566,435 | 565,957 | 44.649 |
| Karbi | KaF07 | 4553331.3 | MK19 | 29,89,46,677 | 658,053 | 657,478 | 16.481 |
| Karbi | KaM03 | 4565523.3 | MK20_1 | 34,63,01,267 | 795,044 | 793,279 | 31.742 |
| Tai-Phake | TPM09 | 4553574.3 | MK21 | 26,99,85,126 | 608,836 | 608,175 | 34.416 |
| Tai-Phake | TPM10 | 4553575.3 | MK22 | 27,45,80,326 | 607,212 | 606,708 | 18.805 |
| Tai-Phake | TPM14 | 4553573.3 | MK23 | 29,01,52,380 | 646,258 | 645,649 | 32.04 |
| Tai-Phake | TPF05 | 4553576.3 | MK24 | 26,40,37,718 | 584,071 | 583,336 | 16.568 |
| Tai-Phake | TPF06 | 4555159.3 | MK25 | 16,45,40,193 | 366,738 | 363,377 | 38.3 |
| Gond | GF12 | 4554736.3 | MK26 | 18,05,47,306 | 404,086 | 403,670 | 50.885 |
| Gond | GM22 | 4554735.3 | MK27 | 18,78,95,595 | 414,468 | 414,016 | 12.164 |
| Gond | GF17 | 4555160.3 | MK28 | 17,34,92,897 | 386,842 | 386,198 | 44.332 |
| Gond | GM26 | 4555161.3 | MK29 | 15,54,69,958 | 345,107 | 344,526 | 39.842 |
| Gond | GF01 | 4565524.3 | MK30_1 | 34,49,10,927 | 792,565 | 790,699 | 32.037 |
| Koya | KF01 | 4555200.3 | MK31 | 14,49,90,779 | 324,067 | 321,411 | 23.202 |
| Koya | KM02 | 4554737.3 | MK32 | 17,50,64,303 | 387,515 | 386,510 | 17.436 |
| Koya | KM27 | 4554738.3 | MK33 | 17,11,87,958 | 381,876 | 380,440 | 51.6 |
| Koya | KF20 | 4554739.3 | MK34 | 23,95,26,156 | 531,070 | 530,546 | 25.998 |
| Koya | KF14 | 4553353.3 | MK35 | 17,36,93,100 | 383,096 | 382,736 | 26.654 |
| Nayak | NM07 | 4553354.3 | MK36 | 16,94,24,715 | 374,035 | 373,224 | 20.357 |
| Nayak | NF17 | 4553364.3 | MK37 | 23,34,58,315 | 519,711 | 519,211 | 44.912 |
| Nayak | NM19 | 4553365.3 | MK38 | 25,86,95,407 | 571,604 | 571,067 | 21.733 |
| Nayak | NM22 | 4553372.3 | MK39 | 26,38,85,597 | 582,571 | 582,060 | 26.234 |
| Nayak | NF15 | 4553373.3 | MK40 | 23,96,11,969 | 535,433 | 535,322 | 37.145 |
| Kolam | MF01 | 4565525.3 | MK41_1 | 31,40,08,249 | 715,632 | 710,822 | 29.927 |
| Kolam | MF03 | 4555203.3 | MK42 | 19,03,36,087 | 420,413 | 420,338 | 63.768 |
| Kolam | MF02 | 4555257.3 | MK43 | 21,28,92,998 | 466,847 | 466,728 | 22.064 |
| Kolam | MM10 | 4555204.3 | MK44 | 17,11,12,094 | 377,416 | 376,790 | 48.493 |
| Kolam | MM11 | 4555258.3 | MK45 | 19,35,38,310 | 430,412 | 430,354 | 28.615 |
| Lepcha | LeM10 | 4555259.3 | MK46 | 18,99,24,800 | 417,353 | 417,310 | 20.409 |
| Lepcha | LeF02 | 4555260.3 | MK47 | 18,11,61,010 | 399,461 | 399,272 | 37.494 |
| Lepcha | LeF04 | 4555261.3 | MK48 | 18,02,76,447 | 395,836 | 395,597 | 11.342 |
| Lepcha | LeM08 | 4555265.3 | MK49 | 16,75,73,928 | 369,324 | 368,021 | 40.071 |
| Lepcha | LeF05 | 4555266.3 | MK50 | 16,38,62,248 | 367,372 | 366,862 | 78.392 |
| Kuki | KuF02 | 4555267.3 | MK51 | 18,40,45,900 | 406,732 | 406,465 | 24.413 |
| Kuki | KuF04 | 4555299.3 | MK52 | 15,02,93,078 | 332,905 | 332,050 | 27.492 |
| Kuki | KuM05 | 4555294.3 | MK53 | 18,20,76,744 | 401,445 | 401,171 | 24.213 |
| Kuki | KuM03 | 4555295.3 | MK54 | 17,50,12,323 | 386,086 | 385,741 | 24.54 |
| Kuki | KuF12 | 4556320.3 | MK55 | 27,97,22,820 | 617,473 | 617,217 | 23.752 |
| Nepali | NeM02 | 4555293.3 | MK56 | 18,98,80,526 | 420,330 | 418,854 | 28.684 |
| Nepali | NeM04 | 4555296.3 | MK57 | 16,57,67,713 | 364,251 | 361,242 | 11.97 |
| Nepali | NeF07 | 4555297.3 | MK58 | 17,71,41,113 | 387,906 | 386,689 | 14.093 |
| Nepali | NeF06 | 4555298.3 | MK59 | 20,62,80,594 | 463,839 | 463,617 | 58.377 |
| Nepali | NeF08 | 4555319.3 | MK60 | 17,98,26,760 | 399,438 | 398,868 | 29.536 |
| Tangkhul | UkF07 | 4555320.3 | MK61 | 18,75,38,433 | 414,123 | 413,067 | 33.583 |
| Tangkhul | UkF04 | 4555321.3 | MK62 | 17,19,47,212 | 379,081 | 377,012 | 20.59 |
| Tangkhul | UkM10 | 4559163.3 | MK63 | 15,98,91,073 | 354,370 | 352,401 | 23.712 |
| Tangkhul | UkM06 | 4556319.3 | MK64 | 16,97,59,133 | 376,081 | 374,163 | 21.006 |
| Tangkhul | UkF02 | 4566265.3 | MK65_1 | 18,79,24,170 | 434,056 | 433,079 | 35.216 |
| Meitei | MeM03 | 4565526.3 | MK66_1 | 16,16,73,662 | 370,226 | 365,644 | 28.015 |
| Meitei | MeF02 | 4566266.3 | MK67_1 | 20,34,38,335 | 467,392 | 466,528 | 19.484 |
| Meitei | MeM06 | 4566267.3 | MK68_1 | 16,10,27,632 | 370,746 | 367,532 | 21.365 |
| Meitei | MeM07 | 4566268.3 | MK69_1 | 18,88,25,869 | 433,533 | 432,408 | 29.754 |
| Meitei | MeF01 | 4555881.3 | MK70 | 17,32,74,524 | 382,900 | 381,604 | 24.059 |
| Bhutia | BuF07 | 4555882.3 | MK71 | 16,33,24,855 | 363,042 | 362,422 | 24.366 |
| Bhutia | BuM01 | 4555883.3 | MK72 | 17,06,23,135 | 376,452 | 374,971 | 38.341 |
| Bhutia | BuF09 | 4555885.3 | MK73 | 18,24,17,445 | 404,167 | 403,445 | 14.47 |
| Bhutia | BuM03 | 4565527.3 | MK74_1 | 27,58,28,776 | 633,886 | 631,651 | 26.138 |
| Bhutia | BuM05 | 4555887.3 | MK75 | 15,96,15,670 | 3,52,695 | 352,163 | 29.124 |

**Table S2** MG-RAST ids of the world population.

| **Population** | **Sample id** | **MG-RAST id** |
| --- | --- | --- |
| Mongolian | 3MG54 | 4556677.3 |
| Mongolian | 3MG57 | 4556680.3 |
| Mongolian | 3MG64 | 4556686.3 |
| Mongolian | 3MG71 | 4556690.3 |
| Mongolian | 3MG75 | 4556693.3 |
| Mongolian | 3MG78 | 4556696.3 |
| Mongolian | 3MG79 | 4556697.3 |
| Mongolian | 3MG80 | 4556698.3 |
| Mongolian | 3MG81 | 4556699.3 |
| Mongolian | 3MG82 | 4556700.3 |
| Mongolian | 3MG83 | 4556701.3 |
| Mongolian | 3MG84 | 4556702.3 |
| Mongolian | 3MG85 | 4556703.3 |
| Mongolian | 3MG86 | 4556704.3 |
| Mongolian | 3MG87 | 4556705.3 |
| Mongolian | 3MG88 | 4556706.3 |
| Mongolian | 3MG89 | 4556707.3 |
| Mongolian | 3MG91 | 4556709.3 |
| Mongolian | 3MG92 | 4556710.3 |
| Mongolian | 3MG93 | 4556711.3 |
| Mongolian | 3MG94 | 4556712.3 |
| Mongolian | 3MG108 | 4556724.3 |
| Hadza | H2 | 4545833.3 |
| Hadza | H3 | 4545842.3 |
| Hadza | H4 | 4545843.3 |
| Hadza | H6 | 4545845.3 |
| Hadza | H7 | 4545846.3 |
| Hadza | H8 | 4545847.3 |
| Hadza | H11 | 4545824.3 |
| Hadza | H13 | 4545826.3 |
| Hadza | H14 | 4545827.3 |
| Hadza | H16 | 4545829.3 |
| Hadza | H17 | 4545830.3 |
| Hadza | H22 | 4545836.3 |
| Hadza | H23 | 4545837.3 |
| Hadza | H27 | 4545841.3 |
| Italian | IT1 | 4545849.3 |
| Italian | IT2 | 4545857.3 |
| Italian | IT3 | 4545858.3 |
| Italian | IT4 | 4545859.3 |
| Italian | IT5 | 4545860.3 |
| Italian | IT6 | 4545861.3 |
| Italian | IT7 | 4545862.3 |
| Italian | IT8 | 4545863.3 |
| Italian | IT12 | 4545852.3 |
| Italian | IT13 | 4545853.3 |
| Italian | IT14 | 4545854.3 |
| Italian | IT15 | 4545855.3 |
| Italian | IT16 | 4545856.3 |
| Amerindian | Amz29adlt | 4489370.3 |
| Amerindian | Amz C10 adlt F1 | 4489396.3 |
| Amerindian | Amz C10 adlt F2 | 4489397.3 |
| Amerindian | Amz C11 adlt F1 | 4489398.3 |
| Amerindian | Amz C11 adlt F2 | 4489399.3 |
| Amerindian | Amz C2 adlt F | 4489422.3 |
| Amerindian | Amz C31 adlt F | 4489427.3 |
| Amerindian | Amz C7 adlt F | 448944.3 |
| Malawian | h130M | 4489829.3 |
| Malawian | h144M | 4489833.3 |
| Malawian | h146M | 4489836.3 |
| Malawian | h147M | 4489839.3 |
| Malawian | h181M | 4489847.3 |
| Malawian | h186M | 4489852.3 |
| Malawian | h209M | 4489860.3 |
| Malawian | h228M | 4489862.3 |
| Malawian | h235M | 4489866.3 |
| Malawian | h257M.2 | 4489869.3 |
| Malawian | h279M | 4489883.3 |
| Malawian | h60M | 4489903.3 |
| Malawian | h9M | 4489922.3 |
| American | TS111 | 4489456.3 |
| American | TS129 | 4489460.3 |
| American | TS15 | 4489463.3 |
| American | TS165 | 4489466.3 |
| American | TS186 | 4489469.3 |
| American | TS195 | 4489472.3 |
| American | TS27 | 4489477.3 |
| American | TS6 | 4489481.3 |
| American | TS9 | 4489484.3 |
| American | Usld Adlt6 | 4489486.3 |
| American | Uschp 18 Mom | 4489496.3 |
| American | Uschp 1 Mom | 4489498.3 |
| American | Uschp 25Mom | 4489500.3 |
| American | Uschp 3Mom | 4489509.3 |

**Table S3** Core gut bacterial genera of the world populations.

| **Core bacterial genus** | **Indian** | **Mongolian** | **Hadza** | **Italian** | **Amerindian** | **Malawian** | **American** |
| --- | --- | --- | --- | --- | --- | --- | --- |
| *Prevotella* | 40.56 | 34.39 |  |  | 29.37 | 30.13 |  |
| *Faecalibacterium* | 11.48 | 11.74 | 21.74 | 27.28 | 14.65 | 16.46 | 22.39 |
| *Eubacterium* | 7.05 | 4.48 | 13.97 | 12.92 | 9.97 | 6.55 | 11.64 |
| *Bacteroides* | 3.03 | 10.97 |  |  | 0.87 | 0.45 | 17.21 |
| *Clostridium* | 4.10 | 5.86 | 12.99 | 4.99 | 8.70 | 8.26 | 18.61 |
| *Blautia* | 1.87 | 1.04 | 10.57 | 12.74 | 3.38 | 2.60 | 4.50 |
| *Ruminococcus* | 1.02 | 3.50 | 2.48 | 7.95 | 3.13 | 3.01 | 20.08 |
| *Roseburia* | 1.97 | 2.00 | 1.39 | 3.44 | 0.22 | 0.55 | 0.37 |
| *Collinsella* | 1.54 | 0.24 |  |  | 0.25 |  |  |
| *Dialister* | 1.37 |  |  |  |  |  |  |
| *Bifidobacterium* |  |  |  | 7.80 |  |  | 13.05 |
| *Bacillus* |  |  |  |  | 1.29 | 1.93 |  |
| *Alistipes* |  | 1.21 |  | 1.47 |  |  | 11.82 |
| *Butyrivibrio* |  |  | 0.27 |  | 2.8 | 2.65 | 4.37 |
| *Treponema* |  |  | 2.75 |  |  |  |  |
| *Butyricicoccus* |  |  | 2.29 | 0.47 |  |  |  |
| *Phascolarctobacterium* |  |  | 0.74 |  | 1.06 |  |  |
| *Erysipelothrix* |  |  | 0.67 |  |  |  |  |
| *Hespellia* |  |  |  |  |  | 1.55 | 3.78 |
| *Lachnospira* |  |  |  | 0.89 |  | 0.45 | 1.04 |
| *Parabacteroides* |  |  |  |  |  |  | 3.47 |
| *Tannerella* |  |  |  |  |  |  | 1.88 |
| *Sphingobacterium* |  |  |  |  |  |  | 1.72 |
| *Barnesiella* |  |  |  |  | 0.64 | 0.62 |  |
| *Selenomonas* |  |  | 1.08 |  |  |  |  |
| *Caloramator* |  |  |  | 0.57 |  |  |  |
| *Desulfonatronovibrio* |  |  | 0.46 |  |  |  |  |

**Table S4** Dietary data of volunteers from Assam, Telangana, Manipur and Sikkim.

| **Serial no.** | **State** | **Location** | **Code** | **Tribe name** | **Gender** | **Age** | **Most common food** | **Fermanted, smoked and dried foods** | **Milk products** |
| --- | --- | --- | --- | --- | --- | --- | --- | --- | --- |
| 1 | Assam | Golaghat | AM01 | Tai- Aiton | Male | 22 | Rice, green leafy vegetables, meat, fish | Fermented bamboo shoot, smoked fish and meat | No |
| 2 | Assam | Golaghat | AM02 | Tai- Aiton | Male | 26 | Rice, green leafy vegetables, meat, fish | Fermented bamboo shoot, smoked fish and meat | Occasionally milk tea |
| 3 | Assam | Golaghat | AM03 | Tai- Aiton | Male | 26 | Rice, green leafy vegetables, meat, fish | Fermented bamboo shoot, smoked fish and meat | Occasionally milk tea |
| 4 | Assam | Golaghat | AM04 | Tai- Aiton | Male | 23 | Rice, green leafy vegetables, meat, fish | Fermented bamboo shoot, smoked fish and meat | Occasionally milk tea |
| 5 | Assam | Golaghat | AM05 | Tai- Aiton | Male | 26 | Rice, green leafy vegetables, meat, fish | Fermented bamboo shoot, smoked fish and meat | Occasionally milk tea |
| 6 | Assam | Golaghat | AM07 | Tai- Aiton | Male | 26 | Rice, green leafy vegetables, meat, fish | Fermented bamboo shoot, smoked fish and meat | Occasionally milk tea |
| 7 | Assam | Golaghat | AF08 | Tai- Aiton | Female | 23 | Rice, green leafy vegetables, meat, fish | Fermented bamboo shoot, smoked fish and meat | No |
| 8 | Assam | Golaghat | AF10 | Tai- Aiton | Female | 22 | Rice, green leafy vegetables, meat, fish | Fermented bamboo shoot, smoked fish and meat | No |
| 9 | Assam | Golaghat | AF11 | Tai- Aiton | Female | 22 | Rice, green leafy vegetables, meat, fish | Fermented bamboo shoot and mastard leaves, smoked fish and meat | No |
| 10 | Assam | Golaghat | AF12 | Tai- Aiton | Female | 23 | Rice, green leafy vegetables, meat, fish | Fermented bamboo shoot, smoked fish | No |
| 11 | Assam | Golaghat | AF13 | Tai- Aiton | Female | 24 | Rice, green leafy vegetables, meat, fish | Fermented bamboo shoot and mastard leaves, smoked fish and meat | No |
| 12 | Assam | Golaghat | AF14 | Tai- Aiton | Female | 23 | Rice, green leafy vegetables, meat, fish | Fermented bamboo shoot and mastard leaves, smoked fish and meat | Occasionally milk tea |
| 13 | Assam | Golaghat | AF15 | Tai- Aiton | Female | 23 | Rice, green leafy vegetables, meat, fish | Fermented bamboo shoot, smoked fish and meat | Milk tea |
| 14 | Assam | Golaghat | AF16 | Tai- Aiton | Female | 22 | Rice, green leafy vegetables, meat, fish | Fermented bamboo shoot and mastard leaves, smoked fish and meat | No |
| 15 | Assam | Golaghat | AF17 | Tai- Aiton | Female | 22 | Rice, green leafy vegetables, meat, fish | Fermented bamboo shoot, smoked fish and meat | No |
| 16 | Assam | Golaghat | AF18 | Tai- Aiton | Female | 22 | Rice, green leafy vegetables, meat, fish | Fermented bamboo shoot, smoked fish and meat | No |
| 17 | Assam | Tinsukia | TPF01 | Tai-Phake | Female | 26 | Rice, dal, fish, meat, green leafy vegetables | Fermented bamboo shoot, fermented fish, fermented mastard leaves | Occasionally milk tea |
| 18 | Assam | Tinsukia | TPF02 | Tai-Phake | Female | 26 | Rice, dal, fish, meat, green leafy vegetables | Fermented bamboo shoot, fermented fish, fermented mastard leaves | Occasionally milk tea |
| 19 | Assam | Tinsukia | TPF03 | Tai-Phake | Female | 23 | Rice, dal, fish, meat, green leafy vegetables | Fermented bamboo shoot, fermented fish, fermented mastard leaves | Milk tea |
| 20 | Assam | Tinsukia | TPF04 | Tai-Phake | Female | 23 | Rice, dal, fish, meat, green leafy vegetables | Fermented bamboo shoot, fermented fish, fermented mastard leaves | Occasionally milk tea |
| 21 | Assam | Tinsukia | TPF05 | Tai-Phake | Female | 25 | Rice, dal, fish, meat, green leafy vegetables | Fermented bamboo shoot, fermented fish, fermented mastard leaves | Occasionally milk tea |
| 22 | Assam | Tinsukia | TPF06 | Tai-Phake | Female | 23 | Rice, dal, fish, meat, green leafy vegetables | Fermented bamboo shoot, fermented fish, fermented mastard leaves | Occasionally milk tea |
| 23 | Assam | Tinsukia | TPM08 | Tai-Phake | Male | 23 | Rice, dal, fish, meat, green leafy vegetables | Fermented bamboo shoot, fermented fish, fermented mastard leaves | Occasionally milk tea |
| 24 | Assam | Tinsukia | TPM09 | Tai-Phake | Male | 26 | Rice, dal, fish, meat, green leafy vegetables | Fermented bamboo shoot, fermented fish, fermented mastard leaves | Occasionally milk tea |
| 25 | Assam | Tinsukia | TPM10 | Tai-Phake | Male | 24 | Rice, dal, fish, meat, green leafy vegetables | Fermented bamboo shoot, fermented fish, fermented mastard leaves | Occasionally milk tea |
| 26 | Assam | Tinsukia | TPM11 | Tai-Phake | Male | 24 | Rice, dal, fish, meat, green leafy vegetables | Fermented bamboo shoot, fermented fish, fermented mastard leaves | Occasionally milk tea |
| 27 | Assam | Tinsukia | TPM13 | Tai-Phake | Male | 24 | Rice, dal, fish, meat, green leafy vegetables | Fermented bamboo shoot, fermented fish, fermented mastard leaves | Occasionally milk tea |
| 28 | Assam | Tinsukia | TPM14 | Tai-Phake | Male | 24 | Rice, dal, fish, meat, green leafy vegetables | Fermented bamboo shoot, fermented fish, fermented mastard leaves | Occasionally milk tea |
| 29 | Assam | Karbi anglong | KaM01 | Karbi | Male | 26 | Rice, dal, vegetables, sour leaves, fish, lentil seeds | Fermented bamboo shoot | No |
| 30 | Assam | Karbi anglong | KaM03 | Karbi | Male | 26 | Rice, dal, green leafy vegetables, meat, fish | Fermented bamboo shoot, smoked fish & meat | No |
| 31 | Assam | Karbi anglong | KaM05 | Karbi | Male | 24 | Rice, dal, seasonal vegetables | Fermented bamboo shoot | Occasionally milk tea |
| 32 | Assam | Karbi anglong | KaF06 | Karbi | Female | 24 | Rice, dal, colocasia with bamboo shoot, vegetables | Fermented bamboo shoot | No |
| 33 | Assam | Karbi anglong | KaF07 | Karbi | Female | 24 | Rice, dal, colocasia with bamboo shoot, vegetables | Fermented bamboo shoot | No |
| 34 | Assam | Karbi anglong | KaF09 | Karbi | Female | 23 | Rice, dal, colocasia, Sour leaves, vegetables, smoked fish & meat. | Fermented bamboo shoot | No |
| 35 | Assam | Karbi anglong | KaM13 | Karbi | Male | 27 | Rice, dal, cereals, green leafy vegetables, fish & meat. | Fermented bamboo shoot | Occasionally milk tea |
| 36 | Assam | Karbi anglong | KaM14 | Karbi | Male | 27 | Rice, dal, pork curry with bamboo shoot, fish & meat. | Fermented bamboo shoot | Occasionally milk tea |
| 37 | Assam | Karbi anglong | KaF15 | Karbi | Female | 22 | Rice, dal, colocasia, sour leaves, bamboo shoot, fish & meat. | Fermented bamboo shoot | Occasionally milk tea |
| 38 | Assam | Karbi anglong | KaF16 | Karbi | Female | 22 | Rice, dal, colocasia, pumpkin, fish & meat. | Fermented bamboo shoot | Occasionally milk tea |
| 39 | Assam | Karbi anglong | KaM17 | Karbi | Male | 23 | Rice, dal, cereals, potato, pumpkin, meat & fish | Fermented bamboo shoot | No |
| 40 | Assam | Karbi anglong | KaM18 | Karbi | Male | 26 | Rice, dal, blackdal, colocasia, meat, fish | Fermented bamboo shoot | No |
| 41 | Assam | Baksa | BF01 | Bodo | Female | 24 | Rice, dal, vegetables, meat, fish | Fermented rice, dry fish, frog, smoked pork | Milk tea |
| 42 | Assam | Baksa | BF02 | Bodo | Female | 24 | Rice, dal, vegetables, meat, fish | Fermented rice, dry fish, frog, smoked pork | Milk tea |
| 43 | Assam | Baksa | BF03 | Bodo | Female | 27 | Rice, dal, vegetables, meat, fish | Fermented rice, dry fish, frog, smoked pork | Milk tea |
| 44 | Assam | Baksa | BF04 | Bodo | Female | 28 | Rice, dal, vegetables, meat, fish | Fermented rice, dry fish, frog, smoked pork | Milk tea |
| 45 | Assam | Baksa | BF05 | Bodo | Female | 27 | Rice, dal, vegetables, meat, fish | Fermented rice, dry fish, frog, smoked pork | Milk tea |
| 46 | Assam | Baksa | BF06 | Bodo | Female | 24 | Rice, dal, vegetables, meat, fish | Fermented rice, dry fish, frog, smoked pork | Milk tea |
| 47 | Assam | Baksa | BF07 | Bodo | Female | 25 | Rice, dal, vegetables, meat, fish | Fermented rice, dry fish, frog, smoked pork | Milk tea |
| 48 | Assam | Baksa | BF08 | Bodo | Female | 24 | Rice, dal, vegetables, meat, fish | Fermented rice, dry fish, frog, smoked pork | Milk tea |
| 49 | Assam | Baksa | BF09 | Bodo | Female | 25 | Rice, dal, vegetables, meat, fish | Fermented rice, dry fish, frog, smoked pork | Milk tea |
| 50 | Assam | Baksa | BF10 | Bodo | Female | 24 | Rice, dal, vegetables, meat, fish | Fermented rice, dry fish, frog, smoked pork | Milk tea |
| 51 | Assam | Baksa | BM11 | Bodo | Male | 24 | Rice, dal, vegetables, meat, fish | Fermented rice, dry fish, frog, smoked pork | No |
| 52 | Assam | Baksa | BM12 | Bodo | Male | 23 | Rice, dal, vegetables, meat, fish | Fermented rice, dry fish, frog, smoked pork | No |
| 53 | Assam | Baksa | BM13 | Bodo | Male | 25 | Rice, dal, vegetables, meat, fish | Fermented rice, dry fish, frog, smoked pork | No |
| 54 | Assam | Baksa | BM14 | Bodo | Male | 25 | Rice, dal, vegetables, meat, fish | Fermented rice, dry fish, frog, smoked pork | Milk tea |
| 55 | Assam | Baksa | BM15 | Bodo | Male | 24 | Rice, dal, vegetables, meat, fish | Fermented rice, dry fish, frog, smoked pork | Milk tea |
| 56 | Assam | Baksa | BM16 | Bodo | Male | 27 | Rice, dal, vegetables, meat, fish | Fermented rice, dry fish, frog, smoked pork | No |
| 57 | Assam | Baksa | BM17 | Bodo | Male | 28 | Rice, dal, vegetables, meat, fish | Fermented rice, dry fish, frog, smoked pork | No |
| 58 | Assam | Baksa | BM18 | Bodo | Male | 23 | Rice, dal, vegetables, meat, fish | Fermented rice, dry fish, frog, smoked pork | Milk tea |
| 59 | Assam | Baksa | BM19 | Bodo | Male | 26 | Rice, dal, vegetables, meat, fish | Fermented rice, dry fish, frog, smoked pork | No |
| 60 | Assam | Baksa | BM20 | Bodo | Male | 25 | Rice, dal, vegetables, meat, fish | Fermented rice, dry fish, frog, smoked pork | No |
| 61 | Assam | Tinsukia | SM01 | Tea Tribe | Male | 26 | Rice, dal, vegetables, meat | Fermented bamboo shoot | No |
| 62 | Assam | Tinsukia | SM02 | Tea Tribe | Male | 24 | Rice, dal, vegetables, meat | Fermented bamboo shoot | Occasionally milk tea |
| 63 | Assam | Tinsukia | SM03 | Tea Tribe | Male | 24 | Rice, dal, vegetables, meat | Fermented bamboo shoot | No |
| 64 | Assam | Tinsukia | SM04 | Tea Tribe | Male | 26 | Rice, dal, vegetables, meat | Fermented bamboo shoot | Occasionally milk tea |
| 65 | Assam | Tinsukia | SM05 | Tea Tribe | Male | 26 | Rice, dal, vegetables | Fermented bamboo shoot | No |
| 66 | Assam | Tinsukia | SM06 | Tea Tribe | Male | 26 | Rice, dal, greenleafy vegetables, meat, fish | Fermented bamboo shoot | Occasionally milk tea |
| 67 | Assam | Tinsukia | SM07 | Tea Tribe | Male | 25 | Rice, dal, vegetables, meat | Fermented bamboo shoot | Rare |
| 68 | Assam | Tinsukia | SF08 | Tea Tribe | Female | 25 | Rice, colocasia, vegetables | Fermented bamboo shoot | Occasionally milk tea |
| 69 | Assam | Tinsukia | SF09 | Tea Tribe | Female | 23 | Rice, vegetables, meat, greenleafy vegetables | Fermented bamboo shoot | No |
| 70 | Assam | Tinsukia | SF10 | Tea Tribe | Female | 24 | Rice, vegetables, meat, greenleafy vegetables | Fermented bamboo shoot | Occasionally milk tea |
| 71 | Assam | Tinsukia | SF11 | Tea Tribe | Female | 23 | Rice, vegetables, meat, fish, greenleafy vegetables | Fermented bamboo shoot | No |
| 72 | Assam | Tinsukia | SF12 | Tea Tribe | Female | 22 | Rice, vegetables, meat, green leafy vegetables | Fermented bamboo shoot | No |
| 73 | Assam | Tinsukia | SF13 | Tea Tribe | Female | 24 | Rice, dal, vegetables | Fermented bamboo shoot | Occasionally milk tea |
| 74 | Assam | Tinsukia | SF14 | Tea Tribe | Female | 23 | Rice, vegetables, fish, meat | Fermented bamboo shoot | No |
| 75 | Assam | Tinsukia | SF15 | Tea Tribe | Female | 24 | Rice, dal, vegetables | Fermented bamboo shoot | Occasionally milk tea |
| 76 | Assam | Tinsukia | SF16 | Tea Tribe | Female | 24 | Rice, dal, vegetables | Fermented bamboo shoot | Occasionally milk tea |
| 77 | Assam | Tinsukia | SF17 | Tea Tribe | Female | 25 | Rice, meat, fish, vegetables | Fermented bamboo shoot | Occasionally milk tea |
| 78 | Assam | Tinsukia | SM18 | Tea Tribe | Male | 25 | Rice, vegetables, meat, green leafy vegetables | Fermented bamboo shoot | No |
| 79 | Telangana | Khammam | NF03 | Nayak | Female | 35 | Rice, pulses, meat, fish | Idli, dosa | Milk tea, curd |
| 80 | Telangana | Khammam | NF04 | Nayak | Female | 35 | Rice, pulses, fish, meat | Idli, dosa | Milk tea, curd |
| 81 | Telangana | Khammam | NM06 | Nayak | Male | 28 | Rice, pulses, fish, meat | Idli, dosa | Milk tea, curd |
| 82 | Telangana | Khammam | NM07 | Nayak | Male | 20 | Rice, pulses, fish, meat | No | Milk tea, curd |
| 83 | Telangana | Khammam | NM08 | Nayak | Male | 20 | Rice, pulses, fish, meat | No | Milk tea, curd |
| 84 | Telangana | Khammam | NM09 | Nayak | Male | 35 | Rice, pulses, fish, meat | No | Milk tea, curd |
| 85 | Telangana | Khammam | NM10 | Nayak | Male | 35 | Rice, pulses, fish, meat | Idli, dosa | Milk tea, curd |
| 86 | Telangana | Khammam | NM11 | Nayak | Male | 28 | Rice, pulses, fish, meat | Idli, dosa | Milk tea, curd |
| 87 | Telangana | Khammam | NM12 | Nayak | Male | 25 | Rice, pulses, fish, meat | Idli, dosa | Milk tea, curd |
| 88 | Telangana | Khammam | NF15 | Nayak | Female | 25 | Rice, pulses, fish, meat | Idli, dosa | Milk tea, curd |
| 89 | Telangana | Khammam | NF17 | Nayak | Female | 23 | Rice, pulses, fish, meat | No | Milk tea, curd |
| 90 | Telangana | Khammam | NM19 | Nayak | Male | 28 | Rice, pulses, fish, meat | Idli, dosa | Milk tea, curd |
| 91 | Telangana | Khammam | NM20 | Nayak | Male | 35 | Rice, pulses, fish, meat | Idli, dosa | Milk tea, curd |
| 92 | Telangana | Khammam | NM21 | Nayak | Male | 35 | Rice, pulses, fish, meat | Idli, dosa | Milk tea, curd |
| 93 | Telangana | Khammam | NM22 | Nayak | Male | 27 | Rice, pulses, fish, meat | Idli, dosa | No |
| 94 | Telangana | Khammam | KF01 | Koya | Female | 20 | Rice, sorghum, roots, fruits, fish, meat | Idli, dosa | Milk tea, curd |
| 95 | Telangana | Khammam | KM04 | Koya | Male | 27 | Rice, sorghum, roots, fruits, fish, meat | No | Milk tea, curd |
| 96 | Telangana | Khammam | KM05 | Koya | Male | 20 | Rice, sorghum, roots, fruits, fish, meat | No | Milk tea, curd |
| 97 | Telangana | Khammam | KM06 | Koya | Male | 22 | Rice, sorghum, roots, fruits, fish, meat | Idli, dosa | Milk tea, curd |
| 98 | Telangana | Khammam | KM07 | Koya | Male | 21 | Rice, sorghum, roots, fruits, fish, meat | Idli, dosa | Milk tea, curd |
| 99 | Telangana | Khammam | KM08 | Koya | Male | 23 | Rice, sorghum, roots, fruits, fish, meat | No | Milk tea, curd |
| 100 | Telangana | Khammam | KF14 | Koya | Female | 28 | Rice, sorghum, roots, fruits, fish, meat | No | Milk tea, curd |
| 101 | Telangana | Khammam | KF18 | Koya | Female | 35 | Rice, sorghum, roots, fruits, fish, meat | No | No |
| 102 | Telangana | Khammam | KF19 | Koya | Female | 20 | Rice, sorghum, roots, fruits, fish, meat | No | Milk tea, curd |
| 103 | Telangana | Khammam | KF23 | Koya | Female | 22 | Rice, sorghum, roots, fruits, fish, meat | No | Milk tea, curd |
| 104 | Telangana | Khammam | KF24 | Koya | Female | 29 | Rice, sorghum, roots, fruits, fish, meat | No | Milk tea, curd |
| 105 | Telangana | Khammam | KM27 | Koya | Male | 25 | Rice, sorghum, roots, fruits, fish, meat | No | Milk tea, curd |
| 106 | Telangana | Khammam | KM28 | Koya | Male | 28 | Rice, sorghum, roots, fruits, fish, meat | No | Milk tea, curd |
| 107 | Telangana | Khammam | KM30 | Koya | Male | 28 | Rice, sorghum, roots, fruits, fish, meat | No | No |
| 108 | Telangana | Khammam | KM31 | Koya | Male | 22 | Rice, sorghum, roots, fruits, fish, meat | No | Milk tea, curd |
| 109 | Telangana | Khammam | KM32 | Koya | Male | 25 | Rice, sorghum, roots, fruits, fish, meat | No | Milk tea, curd |
| 110 | Telangana | Adilabad | MF01 | Kolam | Female | 20 | Rice, ambali, tubers, fish, meat | Idli, dosa | No |
| 111 | Telangana | Adilabad | MF02 | Kolam | Female | 25 | Rice, ambali, tubers, fish, meat | Idli, dosa | No |
| 112 | Telangana | Adilabad | MF03 | Kolam | Female | 23 | Rice, ambali, tubers, fish, meat | Idli, dosa | No |
| 113 | Telangana | Adilabad | MF04 | Kolam | Female | 32 | Rice, ambali, tubers, fish, meat | Idli, dosa | No |
| 114 | Telangana | Adilabad | MF05 | Kolam | Female | 35 | Rice, jowar roti, tubers, fish, meat | Idli, dosa | No |
| 115 | Telangana | Adilabad | MF06 | Kolam | Female | 33 | Rice, jowar roti, tubers, fish, meat | No | No |
| 116 | Telangana | Adilabad | MM07 | Kolam | Male | 23 | Rice, jowar roti, tubers, fish, meat | No | No |
| 117 | Telangana | Adilabad | MM10 | Kolam | Male | 22 | Rice, ambali, tubers, fish, meat | Idli, dosa | No |
| 118 | Telangana | Adilabad | MM11 | Kolam | Male | 22 | Rice, ambali, tubers, fish, meat | No | No |
| 119 | Telangana | Adilabad | MM12 | Kolam | Male | 35 | Rice, ambali, tubers, fish, meat | Idli, dosa | No |
| 120 | Telangana | Adilabad | MM13 | Kolam | Male | 35 | Rice, ambali, tubers, fish, meat | Idli, dosa | No |
| 121 | Telangana | Adilabad | GF01 | Gonds | Female | 27 | Rice, jowar roti, gadaka, ambali, fish, meat | No | No |
| 122 | Telangana | Adilabad | GF04 | Gonds | Female | 35 | Rice, jowar roti, gadaka, ambali, fish, meat | No | Goat milk, curd |
| 123 | Telangana | Adilabad | GF06 | Gonds | Female | 35 | Rice, jowar roti, gadaka, ambali, fish, meat | No | Goat milk, curd |
| 124 | Telangana | Adilabad | GF07 | Gonds | Female | 30 | Rice, jowar roti, gadaka, ambali, fish, meat | No | Goat milk, curd |
| 125 | Telangana | Adilabad | GF08 | Gonds | Female | 32 | Rice, jowar roti, gadaka, ambali, fish, meat | No | Goat milk, curd |
| 126 | Telangana | Adilabad | GF09 | Gonds | Female | 35 | Rice, jowar roti, gadaka, ambali, fish, meat | No | No |
| 127 | Telangana | Adilabad | GF12 | Gonds | Female | 22 | Rice, jowar roti, gadaka, ambali, fish, meat | No | No |
| 128 | Telangana | Adilabad | GF13 | Gonds | Female | 33 | Rice, jowar roti, ambali, fish, meat | No | Goat milk, curd |
| 129 | Telangana | Adilabad | GF14 | Gonds | Female | 32 | Jowar roti, gadaka, ambali, rice, fish, meat | No | Goat milk, curd |
| 130 | Telangana | Adilabad | GF17 | Gonds | Female | 25 | Jowar roti, gadaka, ambali, rice, fish, meat | No | Goat milk, curd |
| 131 | Telangana | Adilabad | GF18 | Gonds | Female | 26 | Jowar roti, gadaka, ambali, rice, fish, meat | Idli, dosa | Goat milk, curd |
| 132 | Telangana | Adilabad | GM19 | Gonds | Male | 32 | Jowar roti, gadaka, ambali, rice, fish, meat | No | No |
| 133 | Telangana | Adilabad | GM20 | Gonds | Male | 35 | Jowar roti, gadaka, ambali, rice, fish, meat | No | No |
| 134 | Telangana | Adilabad | GM26 | Gonds | Male | 25 | Jowar roti, gadaka, ambali, rice, fish, meat | No | Goat milk, curd |
| 135 | Telangana | Adilabad | GM27 | Gonds | Male | 32 | Jowar roti, gadaka, ambali, rice, fish, meat | No | Goat milk, curd |
| 136 | Manipur | Imphal west | MeF01 | Meitei | Female | 25 | Rice, boiled vegetables, meat, fish, bamboo shoot | Ngari (fermented fish), hawaijar (fermented soya bean) | No |
| 137 | Manipur | Imphal East | MeF02 | Meitei | Female | 25 | Rice, boiled vegetables, meat, fish, bamboo shoot | Ngari, hawaijar | No |
| 138 | Manipur | Imphal west | MeF03 | Meitei | Female | 23 | Rice, boiled vegetables, meat, fish, bamboo shoot | Ngari, hawaijar | Milk, milk tea |
| 139 | Manipur | Imphal East | MeF04 | Meitei | Female | 21 | Rice, boiled vegetables, meat, fish, bamboo shoot | Ngari, hawaijar | Milk, milk tea |
| 140 | Manipur | Imphal west | MeF05 | Meitei | Female | 23 | Rice, boiled vegetables, meat, fish, bamboo shoot | Ngari, hawaijar | Milk, milk tea |
| 141 | Manipur | Imphal West | MeM06 | Meitei | Male | 26 | Rice, boiled vegetables, meat, fish, bamboo shoot | Ngari, hawaijar | Milk, milk tea |
| 142 | Manipur | Imphal West | MeM07 | Meitei | Male | 24 | Rice, boiled vegetables, meat, fish, bamboo shoot | Ngari, hawaijar | Milk, milk tea |
| 143 | Manipur | Imphal West | MeM08 | Meitei | Male | 23 | Rice, boiled vegetables, meat, fish, bamboo shoot | Ngari, hawaijar | Milk, milk tea |
| 144 | Manipur | Bishnupur | MeM09 | Meitei | Male | 22 | Rice, boiled vegetables, meat, fish, bamboo shoot | Ngari, hawaijar | Milk, milk tea |
| 145 | Manipur | Bishnupur | MeM10 | Meitei | Male | 20 | Rice, boiled vegetables, meat, fish, bamboo shoot | Ngari, hawaijar | Milk, milk tea |
| 146 | Manipur | Ukhrul | UkF01 | Tangkhul | Female | 21 | Rice, vegetables, meat, fish, bamboo shoot | Ngari, bamboo shoot | Occasionally milk tea |
| 147 | Manipur | Ukhrul | UkF02 | Tangkhul | Female | 22 | Rice, chappati, boiled vegetables, meat, fish | Ngari, hawaijar, bamboo shoot | Occasionally milk tea |
| 148 | Manipur | Ukhrul | UkM03 | Tangkhul | Male | 24 | Rice, boiled vegetables, meat, fish, bamboo shoot | Smoked and Ngari | No |
| 149 | Manipur | Ukhrul | UkF04 | Tangkhul | Female | 27 | Rice, boiled vegetables, meat, fish, bamboo shoot | Ngari, hawaijar, hawaijar | Milk, milk tea |
| 150 | Manipur | Ukhrul | UkF05 | Tangkhul | Female | 26 | Rice, boiled vegetables, meat, fish, bamboo shoot | Smoked andfermented fish | Occasionally milk tea |
| 151 | Manipur | Ukhrul | UkM06 | Tangkhul | Male | 27 | Rice, boiled vegetables, meat, fish, bamboo shoot | Ngari, soy bean, bamboo shoot, smoked fish & meat | Occasionally milk tea |
| 152 | Manipur | Ukhrul | UkF07 | Tangkhul | Female | 26 | Rice, vegetables, meat, fish, bamboo shoot | Smoked and fermented fish | No |
| 153 | Manipur | Ukhrul | UkM08 | Tangkhul | Male | 24 | Rice, vegetables, meat, fish, bamboo shoot | Ngari, soy bean, bamboo shoot, smoked fish & meat | No |
| 154 | Manipur | Ukhrul | UkF09 | Tangkhul | Female | 23 | Rice, vegetables, meat, fish, bamboo shoot | Ngari, soy bean, bamboo shoot, smoked fish & meat | No |
| 155 | Manipur | Ukhrul | UkM10 | Tangkhul | Male | 30 | Rice, vegetables, meat, fish, bamboo shoot | Ngari, soy bean, bamboo shoot, smoked fish & meat | Milk, milk tea |
| 156 | Manipur | Senapati | KuF01 | Kuki | Female | 25 | Rice, vegetables, meat, fish | fermented bamboo shoot | Milk, milk tea |
| 157 | Manipur | Senapati | KuF02 | Kuki | Female | 27 | Rice, vegetables, meat, fish, bamboo shoot | Dried fish, hawaijar, fermented mastard leaves | Milk, milk tea |
| 158 | Manipur | Senapati | KuM03 | Kuki | Male | 20 | Rice, vegetables, meat, fish | Dried fish, fermented mastard leaves | Milk, milk tea |
| 159 | Manipur | Senapati | KuF04 | Kuki | Female | 24 | Rice, vegetables, meat, fish | Dried fish, soy bean, fermented mastard | Milk, milk tea |
| 160 | Manipur | Senapati | KuM05 | Kuki | Male | 20 | Rice, vegetables, meat, fish | Dried fish, fermented bamboo shoot | Milk, milk tea |
| 161 | Manipur | Senapati | KuM06 | Kuki | Male | 20 | Rice, vegetables, meat, fish | Dried fish, fermented bamboo shoot, fermented mastard | Milk, milk tea |
| 162 | Manipur | Senapati | KuM07 | Kuki | Male | 20 | Rice, vegetables, meat, fish | Dried fish, hawaijar, fermented mastard | Milk, milk tea |
| 163 | Manipur | Senapati | KuF09 | Kuki | Female | 20 | Rice, vegetables, meat, fish | Dried fish | Milk, milk tea |
| 164 | Manipur | Senapati | KuM10 | Kuki | Male | 23 | Rice, vegetables, meat, fish | No fermented food | Milk, milk tea |
| 165 | Manipur | Senapati | KuF12 | Kuki | Female | 23 | Rice, vegetables, meat, fish | Dried fish, hawaijar, fermented mastard, bamboo shoot | Milk, milk tea |
| 166 | Sikkim | South Sikkim | NeM01 | Nepali | Male | 26 | Rice, dal, vegetables, fish, meat | Gundruk (fermented mastard leaves), Kenayma (fermented soya bean), Bamboo shoot, smoked meat | Milk, chhurpi, curd, butter |
| 167 | Sikkim | East Sikkim | NeM02 | Nepali | Male | 23 | Rice, dal, vegetables, fish, meat | Gundruk, kenayma, dried meat and smoked meat | Milk, chhurpi, curd, butter |
| 168 | Sikkim | South Sikkim | NeM03 | Nepali | Male | 25 | Rice, dal, vegetables, fish, meat | Gundruk, kinama, bamboo shoot, dried meat and smoked meat | Chhurpi, curd, butter |
| 169 | Sikkim | South Sikkim | NeM04 | Nepali | Male | 25 | Rice, dal, vegetables, fish, meat | Gundruk, kinama, bamboo shoot, dried meat and smoked meat | Chhurpi, curd, butter |
| 170 | Sikkim | West Sikkim | NeF05 | Nepali | Female | 25 | Rice, dal, vegetables, fish, meat | Gundruk, kinama, bamboo shoot, dried meat and smoked meat | Milk, chhurpi, curd, butter |
| 171 | Sikkim | West Sikkim | NeF06 | Nepali | Female | 25 | Rice, dal, vegetables, fish, meat | Gundruk, kinama, bamboo shoot, dried meat and smoked meat | Milk, chhurpi, curd, butter |
| 172 | Sikkim | South Sikkim | NeF07 | Nepali | Female | 24 | Rice, dal, vegetables, fish, meat | Gundruk, kinama, bamboo shoot, dried meat and smoked meat | Milk, chhurpi, curd, butter |
| 173 | Sikkim | South Sikkim | NeF08 | Nepali | Female | 24 | Rice, dal, vegetables, fish, meat | Gundruk, kinama, bamboo shoot, dried meat and smoked meat | Milk, chhurpi, curd, butter |
| 174 | Sikkim | East Sikkim | BuM01 | Bhutia | Male | 24 | Rice, dal, vegetables, fish, meat | Fermented soyabean, bamboo shoot, chilli | Milk, chhurpi, curd, butter |
| 175 | Sikkim | East Sikkim | BuM02 | Bhutia | Male | 23 | Rice, dal, vegetables, fish, meat | Fermented soyabean, bamboo shoot, chilli | Milk, chhurpi,  curd, butter |
| 176 | Sikkim | East Sikkim | BuM03 | Bhutia | Male | 23 | Rice, dal, vegetables, fish, meat | Fermented soyabean, bamboo shoot, chilli | Milk, chhurpi, curd, butter |
| 177 | Sikkim | East Sikkim | BuM04 | Bhutia | Male | 24 | Rice, dal, vegetables, fish, meat | Fermented soyabean, bamboo shoot, chilli | Occasionally milk, churpi |
| 178 | Sikkim | East Sikkim | BuM05 | Bhutia | Male | 23 | Rice, dal, vegetables, fish, meat | Fermented soyabean, bamboo shoot, chilli | Milk, chhurpi, curd, butter |
| 179 | Sikkim | East Sikkim | BuF06 | Bhutia | Female | 24 | Rice, dal, vegetables, fish, meat | Fermented soyabean, bamboo shoot, chilli | Milk, chhurpi, curd, butter |
| 180 | Sikkim | East Sikkim | BuF07 | Bhutia | Female | 27 | Rice, dal, vegetables, fish, meat | Fermented soyabean, bamboo shoot, chilli | Milk, chhurpi, curd, butter |
| 181 | Sikkim | East Sikkim | BuF08 | Bhutia | Female | 27 | Rice, dal, vegetables, fish, meat | Fermented soyabean, bamboo shoot, chilli | Milk, chhurpi, curd, butter |
| 182 | Sikkim | East Sikkim | BuF09 | Bhutia | Female | 26 | Rice, dal, vegetables, fish, meat | Fermented soyabean, bamboo shoot, chilli | Milk, chhurpi, curd, butter |
| 183 | Sikkim | East Sikkim | BuF10 | Bhutia | Female | 28 | Rice, dal, vegetables, fish, meat | Fermented soyabean, bamboo shoot, chilli | Milk, chhurpi, curd, butter |
| 184 | Sikkim | East Sikkim | LeF01 | Lepcha | Female | 26 | Rice, vegetable, pulses, roti | Kenayma, gundruk | Curd, churpi, butter |
| 185 | Sikkim | East Sikkim | LeF02 | Lepcha | Female | 27 | Rice, vegetable, pulses, roti | Kenayma, gundruk | Curd, churpi, butter |
| 186 | Sikkim | East Sikkim | LeF03 | Lepcha | Female | 25 | Rice, vegetable, pulses, roti | Kenayma, gundruk | Curd, churpi, butter |
| 187 | Sikkim | East Sikkim | LeF04 | Lepcha | Female | 24 | Rice, vegetable, pulses, roti | Kenayma, gundruk | Curd, churpi, butter |
| 188 | Sikkim | East Sikkim | LeF05 | Lepcha | Female | 25 | Rice, vegetable, pulses, roti | Kenayma, gundruk | Curd, churpi, butter |
| 189 | Sikkim | North Sikkim | LeM06 | Lepcha | Male | 32 | Rice, vegetable, pulses, roti | Kenayma, gundruk | Curd, churpi, butter |
| 190 | Sikkim | East Sikkim | LeM07 | Lepcha | Male | 23 | Rice, vegetable, pulses, roti | Kenayma, gudruk, smoked meat | Curd, churpi, butter |
| 191 | Sikkim | South Sikkim | LeM08 | Lepcha | Male | 29 | Rice, vegetable, pulses, roti | Kenayma, gudruk, smoked meat, fermented bamboo shoot | Occasionally milk, churpi |
| 192 | Sikkim | West Sikkim | LeM09 | Lepcha | Male | 21 | Rice, vegetable, pulses, roti | Kenayma, gudruk, smoked meat, fermented bamboo shoot | Curd, churpi, butter |
| 193 | Sikkim | North Sikkim | LeM10 | Lepcha | Male | 25 | Rice, vegetable, pulses, roti | Kenayma, gudruk | Curd, churpi, butter |


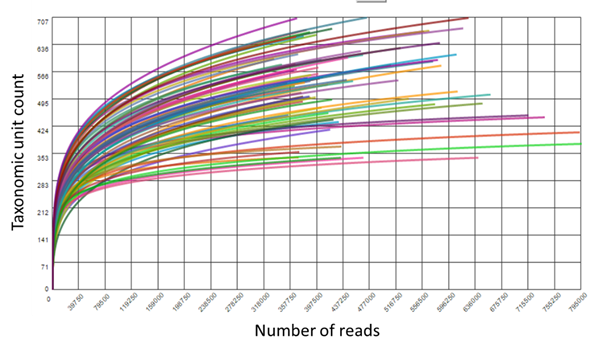


**Figure S1** Rarefaction curve showing the taxonomic unit count against the number of reads obtained for each sample in the next generation sequencing.


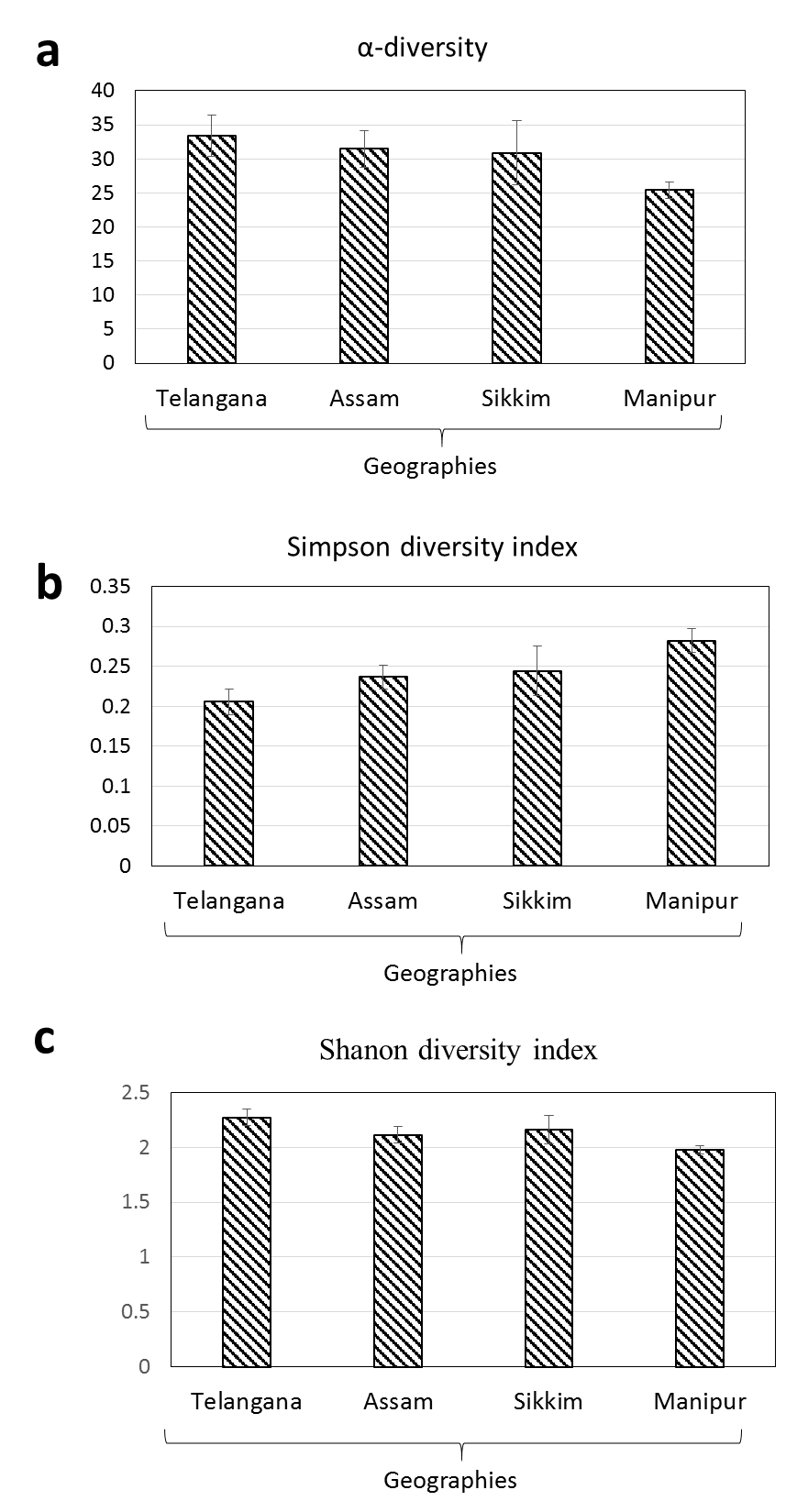


**Figure S2** Bacterial diversity indices across geographies.


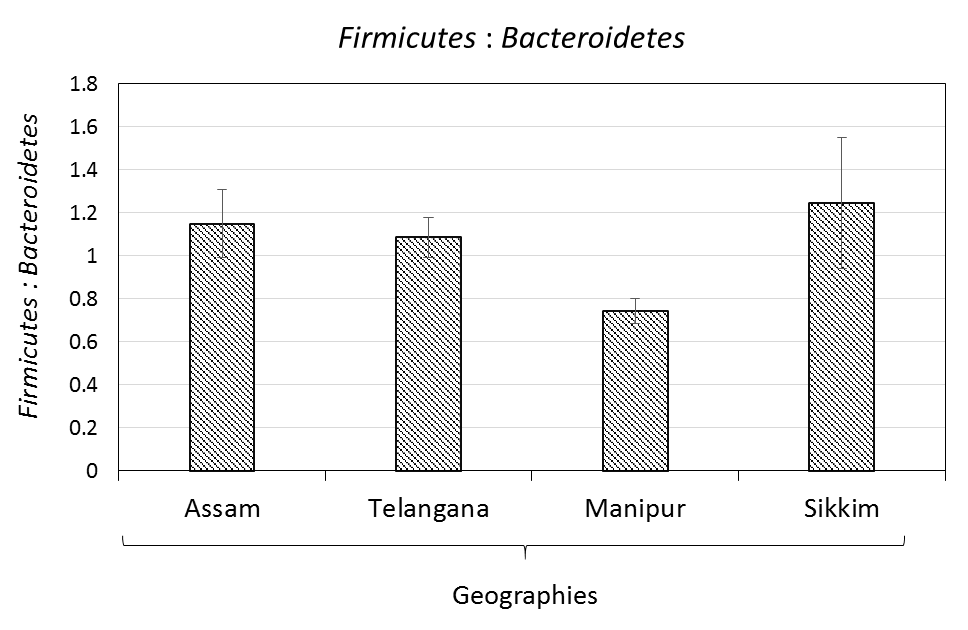


**Figure S3** *Firmicutes* to *Bacteroidetes* (F/B) ratio in the tribes of different geographies.

**Figure S4** *Firmicutes* to *Bacteroidetes* ratio in the tribes of different geographies.
